# The mGluR2 positive allosteric modulator, SAR218645, improves memory and attention deficits in translational models of cognitive symptoms associated with schizophrenia

**DOI:** 10.1038/srep35320

**Published:** 2016-10-13

**Authors:** Guy Griebel, Philippe Pichat, Denis Boulay, Vanessa Naimoli, Lisa Potestio, Robert Featherstone, Sukhveen Sahni, Henry Defex, Christophe Desvignes, Franck Slowinski, Xavier Vigé, Olivier E. Bergis, Rosy Sher, Raymond Kosley, Sathapana Kongsamut, Mark D. Black, Geoffrey B. Varty

**Affiliations:** 1 Sanofi R&D, Strategy, Science Policy & External Innovation, Chilly-Mazarin, France; 2 Sanofi R&D, Translational Sciences Unit, Chilly-Mazarin, France; 3 Sanofi R&D, 1041 Route 202/206, Bridgewater, NJ, USA; 4 Sanofi R&D, Analytical Sciences, Montpellier, France; 5 Sanofi R&D, Lead Generation, Chilly-Mazarin, France

## Abstract

Normalization of altered glutamate neurotransmission through activation of the mGluR2 has emerged as a new approach to treat schizophrenia. These studies describe a potent brain penetrant mGluR2 positive allosteric modulator (PAM), SAR218645. The compound behaves as a selective PAM of mGluR2 in recombinant and native receptor expression systems, increasing the affinity of glutamate at mGluR2 as inferred by competition and GTPγ^35^S binding assays. SAR218645 augmented the mGluR2-mediated response to glutamate in a rat recombinant mGluR2 forced-coupled Ca^2+^ mobilization assay. SAR218645 potentiated mGluR2 agonist-induced contralateral turning. When SAR218645 was tested in models of the positive symptoms of schizophrenia, it reduced head twitch behavior induced by DOI, but it failed to inhibit conditioned avoidance and hyperactivity using pharmacological and transgenic models. Results from experiments in models of the cognitive symptoms associated with schizophrenia showed that SAR218645 improved MK-801-induced episodic memory deficits in rats and attenuated working memory impairment in NMDA Nr1^neo−/−^ mice. The drug reversed disrupted latent inhibition and auditory-evoked potential in mice and rats, respectively, two endophenotypes of schizophrenia. This profile positions SAR218645 as a promising candidate for the treatment of cognitive symptoms of patients with schizophrenia, in particular those with abnormal attention and sensory gating abilities.

Elevated glutamate neurotransmission in the forebrain has been suggested to contribute to the etiology of schizophrenia^1^. Pharmacological activation of the G protein-coupled metabotropic glutamate 2 receptor (mGluR2), which is localized extrasynaptically, including on glutamatergic nerve terminals, has been shown to normalize excessive glutamate levels and increased synaptic activity of glutamate in this region^2^,^3^,^4^,^5^, indicating a viable approach for the treatment of schizophrenia^6^.

In animal studies, mGluR2/3 orthosteric agonists have been shown to produce antipsychotic-like effects in various models (for a review, see ref. 7). For example, LY404039 was found to block phencyclidine (PCP)- and amphetamine-induced hyperactivity and affect conditioned avoidance behavior in rodents^8^. The finding that the effects of mGluR2/3 agonists in the hyperactivity models was lost in mGluR2, but not in mGluR3 knockout mice, indicated that the mGluR2 rather than the mGluR3 is responsible for the antipsychotic-like activity of these drugs^9^,^10^. While these findings point towards a beneficial role of mGluR2/3 agonists against the positive symptoms of schizophrenia, other studies in rodents have indicated that these compounds may have an additional potential to treat the cognitive symptoms of this condition, although the results have been inconsistent throughout the studies (see ref. 7 for a review of these findings). The efficacy of the orthosteric mGluR2/3 agonist, LY2140023, has been evaluated in schizophrenia patients. While the initial trial demonstrated efficacy in the treatment of positive and negative symptoms^11^, an additional study failed to replicate these findings. Importantly, this second study reported an increase risk of seizures associated with LY2140023 treatment^12^.

Positive allosteric modulators (PAMs) of mGluR2 that interact with a site topographically distinct from the orthosteric glutamate site, may offer an alternative approach to nonselective mGluR2/3 agonists^13^,^14^. As the orthosteric binding site is highly conserved between mGluR2 and mGluR3, it is difficult to achieve high selectivity for an individual receptor with a direct agonist, unlike PAMs of these receptor subtypes. Moreover, PAMs, which have minimal effect on their own, require the synaptic release of the neurotransmitter and the activation of the receptor by the endogenous agonist, limiting the risk of receptor over-activation and the development of tolerance through desensitization following protracted dosing as seen with orthosteric mGluR2/3 agonists^9^,^10^,^15^,^16^,^17^.

Highly selective PAMs of the mGluR2 with structural diversity have been identified^14^,^18^. They have been reported to display antipsychotic-like activity in various rodent models, including psychostimulant-induced hyperlocomotion or disruption of prepulse inhibition of the acoustic startle reflex, and conditioned avoidance behavior^15^,^19^,^20^,^21^,^22^,^23^,^24^. Based on these findings, mGluR2 PAMs have entered into clinical trials. In an exploratory study in stably treated patients with schizophrenia, the mGluR2 PAM, JNJ-40411813, was found to attenuate ketamine-induced increases in Brief Psychiatric Rating Scale scores mostly *via* an effect on negative symptoms^25^. Together, these findings suggest that mGluR2 PAMs may offer a beneficial therapeutic approach for the treatment of schizophrenia.

Here, we report the detailed characterization of a structurally distinct, highly potent and selective mGluR2 PAM, here named SAR218645 ((S)-2-(1,1-dimethyl-indan-5-yloxymethyl)-2,3-dihydro-oxazolo[3,2-a]pyrimidin-7-one) (Fig. 1). Specifically, the therapeutic potential of SAR218645 was evaluated in various animal models addressing different symptoms of schizophrenia, including hallucinations (psychotomimetic-induced head-twitch behavior), abnormal motor behavior (psychotomimetic-induced motor activity in normal mice and spontaneous hyperactivity in transgenic mice), cognitive deficits (psychotomimetic-induced impairment in visual episodic and working memory), and abnormalities in sensory processing and attention (amphetamine-induced disruption in latent inhibition and auditory evoked potential). Moreover, we suggest a possible translational approach for initial clinical studies.

## Materials and Methods

### Ethics statement

All experimental procedures described herein were carried out in accordance with the “Guide and Care and Use of Laboratory Animals” (National Institutes of Health) and approved by the Sanofi Institutional Animal Care and Use Committee (studies conducted in the USA) or the Animal Ethics Committee of Sanofi (studies conducted in France).

### Animals

Animals had access to food and water ad libitum with a 12-h light/dark cycle (lights on at 7:00 a.m.). The following species and strains were used: (1) mice: CD-1, C57BL/6J, OF1 and Swiss (Charles River Laboratories, US; Janvier Labs, Le Genest Saint Isle, France); (2) Rats: Sprague-Dawley (Charles River Laboratories, France) (see below for further details). All testing was performed during the light (day) cycle.

### Drugs

Diazepam, LY341495, LY404039, SAR218645, SSR180711 and volinanserin (Sanofi Medicinal Chemistry), levetiracetam (Advanced Technology & Industrial Co. Ltd, Hong Kong), pentylenetetrazol, MK-801 (Sigma RBI, St Quentin Fallavier, France), haloperidol (Bell Medical Services, Inc., Marlborough, NJ), clozapine (Tocris Bioscience, Bristol, UK), olanzapine (Interchim, Clichy, France), amphetamine, 2,5-dimethoxy-4-iodoamphetamine [(±)-DOI] hydrochloride, L-glutamatic acid (Sigma-Aldrich, US or France), were dissolved or suspended in distilled water with 0.6% methylcellulose and the addition of 5% Tween 80 (Sigma RBI) (unless otherwise indicated) in *in vivo* studies and suspended in DMSO at 10 mM in *in vitro* experiments. Doses refer to the weight of the free base. Volume of administration was 10 or 20 ml/kg in mice, 2 or 5 ml/kg in rats. All drug solutions were prepared fresh daily.

## Synthesis of SAR218645

**Figure 1:**
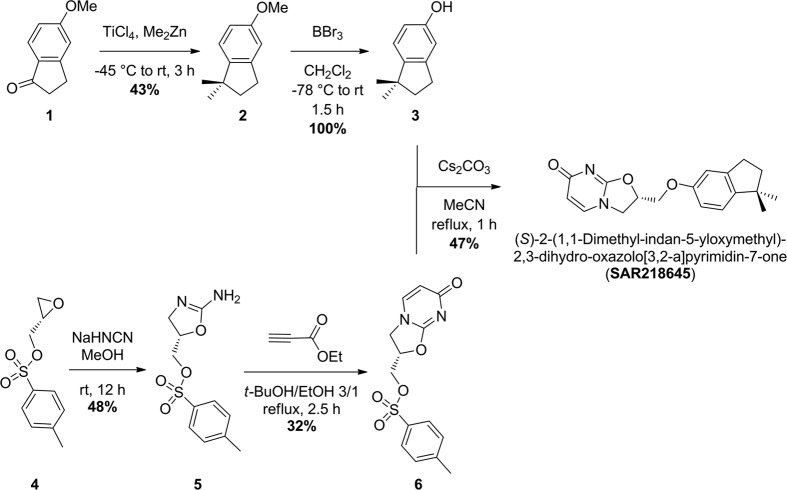
Synthesis of SAR218645. SAR218645 was prepared in 47% yield *via* the substitution of (*S*)-2-toluene-4-sulfonic acid methyl-2,3-dihydro-oxazolo[3,2-a]pyrimidin-7-one (6) by 1,1-dimethyl-indan-5-ol (3). Compounds (**3**) and (**6**) were prepared in two steps from respectively, 5-methoxy-1-indanone (**1**) (in 43% overall yield) and from (2*S*)-glycidyl tosylate (**4**) (in 15% overall yield).

The synthesis of SAR218645 was achieved as depicted in Fig. 1. 1,1-Dimethyl-indan-5-ol (**3**) was prepared *via* a two step sequence reported by Inoue *et al*.^26^ as follows: 5-methoxy-1-indanone (**1**, 2.0 g) was converted into 5-methoxy-1,1-dimethylindane (**2,** 0.941 g) in 43% yield by dropwise addition of **1** (2.0 g) in 15 ml of anhydrous dichoromethane to a mixture of titanium tetrachloride (4.91 g) and dimethyl zinc (25.6 mL of a 1.01 M toluene solution) in anhydrous dichloromethane (30 mL) at −45 °C. The mixture was then allowed to warm to room temperature and stirred at room temperature for 3 h. The mixture was then poured into ice water and the resulting aqueous solution extracted three times with ethyl acetate. The combined ethyl acetate extract was washed with brine and dried over sodium sulfate and filtered. The solution was then concentrated under reduced pressure and the residue purified by flash chromatography on silica gel to provide 0.941 g (43%) of compound **2**. Compound **2** (0.941 g) was then converted into 1,1-dimethylindan-5-ol (**3**, 0.878 g) in quantitative yield: To a stirred solution of compound **2** (0.941 g) in 20 ml of anhydrous dichloromethane at −78 °C was added dropwise boron tribromide (10.7 mL of a 1.0 M solution) in dichloromethane. The mixture was warmed from −78 °C to room temperature and stirred for 1.5 h at room temperature. The mixture was then quenched by slow addition of 15 ml of water and extracted with dichloromethane. The combined organic extract was washed with brine, dried over sodium sulfate and filtered. Concentration of the resulting solution under reduced pressure provided 0,878 g of **3**. Analysis of 1,1-dimethylindan-5-ol (**3**): ^1^H nuclear magnetic resonance (300 MHz in CDCl_3_): 6.98 (d, 1H), 6.66 (s, 1H), 6.64 (d, 1H), 4.51 (s, 1H), 2.83 (t, 2H), 1.92 (t, 2H), 1.23 (s, 6H). LC-MS ESI m/z 163.11 [C_11_H_15_O (M + H) requires 163.10].

In parallel, (*S*)-2-toluene-4-sulfonic acid methyl-2,3-dihydro-oxazolo[3,2-a]pyrimidin-7-one (**6**) was synthesized in two steps following the procedure described by Cao *et al*.^27^: (2*S*)-glycidyl tosylate (**4**, 10 g) in methanol was added to a solution of sodium hydrogen cyanamide (2.81 g) in methanol (44 mL) and the resulting mixture was stirred at room temperature overnight. Then methanol was removed by concentration *in vacuo* and the resulting residue was diluted with water and ethyl acetate. The organic layer was then dried over sodium sulfate, and concentrated *in vacuo* to provide 5.74 g (48%) of (*S*)-5-(toluene-4-sulfonic acid methyl)-(4,5-dihydro-oxazol-2-yl)amine (**5**). To a solution of (**5**, 5.69 g) in a 3/1 *tert*-butanol/ethanol solution (150/50 mL) was added ethyl propiolate (2.14 mL) and the resulting mixture stirred for 2.5 h under reflux. Then the mixture was cooled to room temperature, concentrated *in vacuo*, and the resulting residue was purified by flash chromatography to furnish 2.15 g (32%) of (*S*)-2-toluene-4-sulfonic acid methyl-2,3-dihydro-oxazolo[3,2-a]pyrimidin-7-one (**6**). ^1^H nuclear magnetic resonance (300 MHz in DMSO-*d*_*6*_): 7.80 (d, 2H), 7.69 (d, 1H), 7.51 (d, 2H), 5.79 (d, 1H), 5.24–5.15 (m, 1H), 4.43–4.26 (m, 2H), 4.27 (t, 1H), 3.90 (dd, 1H), 2.05 (s, 3H). LC-MS ESI m/z 323.07 [C_14_H_15_N_2_O_5_S (M + H) requires 323.06].

Finally, SAR218645 was obtained *via* the substitution of (*S*)-2-toluene-4-sulfonic acid methyl-2,3-dihydro-oxazolo[3,2-a]pyrimidin-7-one (**6**, 0.8 g) by 1,1-dimethyl-indan-5-ol (**3**, 0.4 g) in anhydrous acetonitrile (80 mL) in the presence of cesium carbonate (0.82 g) under reflux for 1 h^28^. After 1 h, the whole mixture was concentrated, the resulting residue diluted with water and extracted with ethyl acetate. The combined organic layers were washed successively with 3% HCl, an aqueous saturated solution of NaHCO_3_, brine and finally dried over sodium sulfate. Flash chromatography on silica gel led to 0.4 g (47% yield) of the title compound, SAR218645. ^1^H nuclear magnetic resonance (300 MHz in CDCl_3_): 7.24 (d, 1H), 7.03 (d, 1H), 6.72 (s, 1H), 6.69 (d, 1H), 6.08 (d, 1H), 5.27 (m, 1H), 4.20–4.41 (m, 4H), 2.84 (t, 2H), 1.92 (t, 2H), 1.23 (s, 6H). [α]_D_^25^ = −55.0 (c = 1.11, CHCl_3_). LC-MS ES + m/z 313.14 [C_18_H_21_N_2_O_3_ (M + H) requires 313.14].

## Functional *in vitro* mGluR2 assays

### Ca^2+^ mobilization assays

Recombinant rat and human mGluR2 receptors were stably expressed in HEK293 Flp-in-T-REx cell lines with Gα16. Receptor and G-protein expression were induced with either tetracycline or doxycycline 20–24 hr before the experiment. Growth medium was replaced with HBSS buffer containing Ca^2+^ and Mg^2+^, 20 mM HEPES. Calcium mobilization was monitored using Fluo-4 or Calcium-3 dye on a Functional Drug Screening System (FDSS) reader (Hamamatsu Photonics, Hamamatsu City, Japan). Responses were measured as peak fluorescence intensity minus basal fluorescence. EC_50_ values were calculated using 4-parameter curve fitting. For the measurement of intrinsic agonism, SAR218645 was added alone (i.e., in the absence of glutamate); for measurement of PAM activity, SAR218645 was added together with an EC_10_ (rat)- or EC_20_ (human)-equivalent concentration of glutamate. The subtype selectivity of SAR218645 for mGluR2 versus other metabotropic receptors was determined using a Multispan Allosteric Profiling panel (Multispan, Inc., Hayward, CA, USA).

### Measurement of cAMP accumulation

Recombinant human mGluR2 receptor and the excitatory amino acid transporter SLCA1 were stably expressed in a doxycycline inducible HEK293 Flp-in-T-Rex cell line. Growth medium was replaced with HBSS containing Ca^2+^ and Mg^2+^, 20 mM HEPES. Cells were treated with the compounds, and cAMP production was induced with 1 μM Forskolin for 5 min. cAMP was detected using the CisBio HTRF detection kit. Potentiator responses were normalized to 100 μM glutamate and 1 μM Forskolin. EC_50_ were calculated using 4-parameter curve fitting.

### [^35^S]GTPγS binding to mouse cortex

The assay was performed in 96-well filtration plates in 250 μl total volume. 20 μg/ml of membranes were incubated in 20 mM HEPES, 100 mM NaCl, 3 mM MgCl_2_, 0.1 mM EGTA, 3 μM GDP, pH 7.4 buffer with 0.5 nM [^35^S]GTPγS, glutamate and SAR218645 for 1 hr at room temperature. For termination of the reaction, the plate was filtered and dried prior to adding 50 μl Microscint 40, and counted on the 1450 TopCount Microbeta Counter. EC_50_ were calculated using 4-parameter curve fitting.

### [^3^H]LY341495 Binding

Membranes were prepared from an inducible HEK-293 cell line expressing rat mGluR2 or mGluR3. Binding assay was adapted from Johnson *et al*.^29^. Briefly, [^3^H]LY341495 (1.4 nM) was evaluated for binding in the presence or in the absence of orthosteric and allosteric ligands. Nonspecific binding was determined in the presence of 2 mM glutamate. Addition of 5–10 μg membrane protein initiated the incubation. After 45 min at room temperature, the incubation was terminated by rapid filtration followed by scintillation counting. Ki’s were calculated using 4-parameter curve fitting.

### *In vitro* selectivity profile

The effect of SAR218645 on approximately 180 different receptors, ion channels, enzymes, transporters and kinases (see Supplementary Material Table S1) was evaluated at a contract research organization (CEREP, Celle L’Evescault, France) using established protocols or through internal studies. IC_50_ were determined in cases where significant activity was observed at 10 μM (≥50% inhibition).

### Plasma exposure and brain penetration

Plasma and brain exposures of SAR218645 in male CD-1 mice (25–30 g at arrival) were measured after single oral doses of 1, 3 and 10 mg/kg. Mouse plasma samples (25.0 μl) were transferred into micro-centrifuge tubes (1.7 ml) and mixed with 50 μl of Internal Standard solution (500 ng/ml in acetonitrile). The tubes were vortex mixed for 1 min and centrifuged at 10,000 g for 5 min. An aliquot of the supernatant (50 μl) was transferred into an autosampler vial and mixed with 100 μl of water for LC/MS/MS analysis. Each mouse brain was first homogenized with 3.0 ml of 25% acetonitrile in water. The tissue homogenates (50.0 μl) were transferred into micro-centrifuge tubes (1.7 ml) and mixed with 100 μl of Internal Standard solution (500 ng/ml of A000597492 in acetonitrile). The tubes were vortex mixed for 1 min and centrifuged at 10,000 g for 5 min. An aliquot of the supernatant (50 μl) was transferred into an autosampler vial and mixed with 100 μl of water for LC/MS/MS analysis. The lower limits of quantitation (LLOQ) for SAR218645 were 0.500 ng/ml for plasma and 0.100 ng/ml for brain homogenate. The calibration curves were linear in the above prepared concentration ranges. The LLOQ for brain tissue was estimated to be approximately 0.80 ng/g. The pharmacokinetic parameters were calculated from the arithmetic mean of the group sample concentrations using the program WinNonLin 5.2., non-compartment model 200 for the extravascular routes.

## Functional *in vivo* assay

### LY404039-induced turning behavior in mice

Under light restraint, a single unilateral intracortical infusion of the selective mGluR2/3 receptor agonist, LY404039, was performed in CD-1 mice (25–30 g at arrival). An injection point was assessed using the eyes and back of the skull as landmarks. Immediately following the application of LY404039, the observation period began. The number of contralateral turns elicited in a 5 min period was recorded. SAR218645 was administered orally 60 min before infusion of LY404039 and the potentiation of the turning behavior was noted. The selective mGluR2/3 receptor antagonist, LY341495, was administered ip 90 min prior to infusion of LY404039. Statistical analysis was performed using Kruskal Wallis test followed by a Wilcoxon two-tailed comparison. Ten animals per group were used.

## Assessment of mGluR2-5-HT_2A_ receptor interaction

### DOI-induced glutamate release in the rat medial prefrontal cortex

Male Sprague-Dawley rats (320–350 g) were housed two per cage. Two days before the dialysis assay, they were anesthetized with chloral hydrate (400 mg/kg, ip, 10 ml/kg of body weight) and placed in a stereotaxic apparatus (David Kopf Instruments, Tujunga, CA, USA). Anesthesia was maintained throughout surgery as necessary with supplementary doses of chloral hydrate. Body temperature was monitored by a rectal probe and adjusted (37 ± 1 °C) by a homeothermic blanket. The skull and the dura were opened to allow the implantation of a guide cannula in the medial prefrontal cortex (PFC). The coordinates were 2.5 mM anterior to bregma, 0.6 mM lateral to the midline and 1.3 mM below the dural surface^30^. A dental cement cap held the cannula in place, and three screws anchored the cap to the skull. The rats were individually housed post-surgery and allowed two days of recovery before the start of the experiment. On the day of the experiment, animals were placed in a microdialysis bowl, the cannula cap was removed, and a 3 mM microdialysis probe (CMA12, Carnegie Medicine AB, Stockholm, Sweden) was inserted into the guide cannula. The probe was perfused at a constant flow rate of 1 μl/min using a microinjection pump (CMA100; Carnegie Medicine AB) with a gassed Ringer’s solution containing (in mM): NaCl (145), KCl (2.7), CaCl_2_ (1.2), MgCl_2_ (1), Na_2_HPO_4_ (2.3), Na_2_HPO_4_ (0.45); pH 7.4. Microdialysis sampling started 120 min after probe placement into the PFC. The outlet of the probe was connected to an on-line derivatization system allowing direct analysis of dialysate samples collected every 15 min. Glutamate levels were measured in 15 μl dialysate samples using capillary electrophoresis (CE) with laser-induced fluorescence detection. Before analysis, the samples were derivatized using naphtalene-2,3-dicarboxaldehyde and sodium cyanide, as previously described^31^. CE experiments were performed on a P/ACE MDQ capillary electrophoresis system (Beckman Coulter, Villepinte, France) coupled to an external Zetalif fluorescence detector (Picometrics S.A., Toulouse, France). The excitation was performed by an Omnichrome (Melles Griot Laser Products) helium-cadmium laser at a wavelength of 442 nm with a 30 mW excitation power. The emission intensity was measured at a wavelength of 490 nm. Separations were carried out with a fused-silica capillary (Polymicro Technology, Phoenix, AZ, USA) of 50 μm i.d. and 375 μm o.d. having a total length of 55 cm and an effective length of 38.9 cm with an applied voltage of 25 kV (i.e., 65 μA current). Borate buffer (75 mM) containing β-Cyclodextrin (1 mM), pH 10.5, was used for CE running. At the end of the experiments, an injection of sky blue solution was performed through the probe and animals were sacrificed with an overdose of pentobarbital. The brain was removed, frozen, and 50 μm thick sections were cut with a cryostat to verify correct placement of the microdialysis probe. Glutamate levels in fractional samples were converted to a percentage of the mean value of the 90 min baseline measurements before treatment. Time-course effect of SAR218645 on glutamate levels was analyzed by two-way ANOVA with treatment as a between-subjects factor and time of sampling as a within-subjects factor, followed by Dunnett’s post-hoc tests. Dose-effect of SAR218645 was evaluated by comparing the area under the curve during the first 150 min after po injection of the drug or vehicle. SAR218645 (0.3, 1, 3 or 30 mg/kg po) or its vehicle (Tween80 5%/CMC 0.6%) were administered 30 min before the 5-HT_2A_ receptor agonist, DOI (2.5 mg/kg sc) or its vehicle. Statistical analysis was carried out by one-way ANOVA followed by Dunnett’s post-hoc tests. Five to nine animals per group were used.

## Methods: Effects of SAR218645 in models of schizophrenia-related symptoms

### Models predictive of therapeutic activity against positive symptoms

#### DOI-induced head-twitch behaviors in mice

Male CD-1 mice weighing 25–30 g at the time of testing were used. Animals were brought into the testing area and allowed at least 30 min to acclimatize. The behavior observation was conducted in a wire test chamber measuring 10 cm^3^. SAR218645 or the selective 5-HT_2A_ receptor antagonist, volinanserin, was administered po at various times before administration of DOI. DOI was injected ip at a dose of 1 mg/kg. Five minutes later behavioral observation commenced. A trained (blinded) observer continuously watched the mice for 20 min and noted the number of DOI-induced head twitches during this time. The number of head twitches per group was averaged and SAR218645 or volinanserin plus DOI were compared to DOI alone. Three to eight animals per group were used.

#### Locomotor hyperactivity in wild-type and transgenic mice

An actimeter device consisting of a cylinder (20 cm diameter, 9.5 cm height, Apelex, France) equipped with two perpendicular light beams located 1.5 cm above the floor was used. Mice from the transgenic batches (DAT^−/−^ and NMDA Nr1^neo−/−^) were orally pretreated with either SAR218645 or vehicle and individually isolated in boxes for a period of 60 min. Animals were then placed in the actimeter device and locomotor activity (number of interrupted light beams) was recorded for a period of 30 or 60 min. Mice in which the expression of the dopamine transporter (DAT) was knocked out (i.e., the DAT^−/−^ mouse) have been proposed as a reliable model of the positive symptoms of schizophrenia^32^. In these mice, dopamine levels in the synapse are dramatically elevated and these animals are hyperactive and agitated in behavioral tests. NMDA Nr1^neo−/−^ mice express only 5 to 10% of normal levels of the NR1 subunit of the NMDA receptor^33^, thus mimicking a hypo-glutamatergic state. These mice exhibit behavioral abnormalities (impairments in habituation, sensorimotor gating and social behavior, and hyperactivity), which closely resemble those observed following pharmacological blockade of the NMDA receptor. As such, they have been proposed as an animal model of schizophrenia^33^,^34^,^35^,^36^. DAT^−/−^ and NMDA Nr1^neo−/−^ mice did not show spontaneous stereotyped behaviors. As such, in our present experiments, a relatively moderate dose of amphetamine (2 mg/kg ip) or MK-801 (0.2 mg/kg, ip) was used to selectively induce similar hyperactivity, with no noticeable appearance of stereotyped behaviors in normal mice. For amphetamine or MK-801 antagonism experiments, male Swiss mice (20–30 g) were orally pretreated with SAR218645, haloperidol or vehicle, immediately individually isolated in a Plexiglas box, followed 30 min later by administration of vehicle, amphetamine or MK-801. Immediately following amphetamine challenge, 30 min after MK-801 challenge, mice were placed in the actimeter devices and locomotor activity was recorded for a period of 30 (DAT^−/−^) or 60 (NMDA Nr1^neo−/−^) min.

Statistical analyses were performed using SAS V8.2 software (SAS Institute, Cary, NC, USA). The number of light beam breaks (motility count) recorded for 30 or 60 min were analyzed using one-way ANOVAs using a fixed factor of challenge or genotype and complementary post hoc (Newman-Keuls) tests were performed. Five to nine animals per group were used.

#### Conditioned active avoidance response (CAR) in mice

CAR behavior was assessed using automated two-way shuttle boxes. C57BL/6J mice (29–40 g) were trained for 3 days-a-week to move into the adjacent compartment (avoidance) within 15 s upon presentation of the conditioned stimulus (light) in order to avoid the delivery of an unconditioned stimulus (0.4 mA scrambled electric foot shock of 20 s maximum). Shuttle responses performed during the 15-s avoidance period were recorded as avoidance responses and no shock was received. Each daily test session consisted of 40 trials. SAR218645 and olanzapine were administered po 60 min prior to testing. Statistics performed on total avoidances consisted of using a one-way ANOVA, followed by a post-hoc Dunnett’s test for individual comparisons. Eight animals per group were used.

### Models predictive of therapeutic activity against the cognitive symptoms

#### The novel object recognition task in mice

The test apparatus was based on that described by Ennaceur and Delacour^37^ in rats and adapted for use in mice^38^. The apparatus consisted of a uniformly lit (20 lux) PVC enclosure (52 L × 52 W × 40 H cm) with a video camera positioned 160 cm above the bench. The objects to be discriminated were a metal triangle (3.3 cm height, 5.5 cm wide) and a plastic piece of construction game (3 cm height and 3 cm wide). The observer was located in an adjacent room fitted with a video monitoring system. The experiment consisted of 3 sessions. During the first session, male Swiss mice (25–30 g) were allowed to become familiar with the experimental environment for 5 min (S1). Time spent active (animal moving around with or without sniffing and exploration) was measured. Twenty-four hours later, the animals were placed in the same enclosure containing two identical objects for the amount of time necessary to spend 20 s exploring these two objects to a limit of 5 min (exploration was defined as the animal having its head within 2 cm of the object while looking at, sniffing, or touching it) (S2). After a ‘forgetting’ interval of 60 min, mice were placed back into the enclosure with a previously presented familiar object and a novel object for a period of 5 min (S3 or recall session). Times spent exploring the familiar and novel objects were recorded. Following a 60 min ‘forgetting’ interval, normal mice spent more time exploring the novel object compared to the familiar one during S3. This reflects the ability of the animal to remember the familiar object. Short-term memory was impaired by MK-801 injection (0.125 mg/kg ip), resulting in mice spending the same amount of time exploring both objects, reflecting a forgetting of the familiar object and a short-term visual memory deficit. Both MK-801 and SAR218645 were administered once immediately after S2. Data (time exploring each of the two objects, in seconds) were analyzed with a two-way ANOVA, with the treatment and the object as the between factors, followed by a Winer analysis for comparing the time spent exploring the familiar versus the novel object for each treatment. Eight to ten animals per group were used.

#### The Y-maze test in NMDA Nr1^neo−/−^ mice

Pilot experiments have indicated that NMDA Nr1^neo−/−^ mice display impaired short-term memory in the Y-maze. The apparatus consisted of 3 arms in gray PVC in the shape of a Y. Arms were 28 cm long, 6 cm wide with walls 15 cm high. Movement was tracked manually using in-house software by an experimenter located in an adjacent room via a camera mounted directly above the maze. The animal was placed in an arm facing the center (Arm A) for 5 min. A correct alternation occurred when the animal moved to the other 2 arms without retracing its steps (i.e. Arm A to B to C). Movements such as ABA were incorrect. Based on the movement over the entire session, the percentage of correct alternations was calculated [i.e., (Total number of alternations × 100)/(Total number of arm entries-2)]. SAR218645 was administered po at 0.1 mg/kg 60 min prior to testing to either wild-type or NMDA Nr1^neo−/−^ mice. No dose-response effect was investigated due to the limited number of transgenic animals available. Statistics performed on total arm entries and percentage alternation consisted of using a two-way ANOVA, followed by a post-hoc Winer test for individual comparisons. Student’s *t*-test was used to compare the performance of each group with the chance level. Ten to eleven animals per group were used.

#### Amphetamine-induced disruption of latent inhibition (LI) in mice

The procedure was based on that described by Lipina *et al*.^39^. Briefly, male C57BL/6J mice (20–30 g, 6–8-week-old) were water restricted and trained to drink in an operant chamber for 15 min over 5 daily sessions. The LI procedure was completed in the following stages each given 24 h apart: (*a*) *Pre-exposure*: The preexposed (PE) animals received 40, 85-dB white noise presentations in the chamber for 50 min; the non-preexposed (NPE) animals were confined to the chamber for the same period but without the presence of the tone. (*b*) *Conditioning*: All mice received 2 tone-shock (1 s, 0.37 mA) pairings in the chamber. (*c*) *Test*: Each mouse was placed in the chamber and allowed to drink. After completing 75 licks, the white noise was presented and lasted until the mouse completed 100 licks. For data analysis, the time during 50–75 licks (A period) and the time to complete licks 76–100 (after tone onset; B period) was calculated into a suppression ratio defined as A/(A+B). A low suppression ratio indicates a stronger suppression of drinking. LI is defined as an increase in suppression ratio (little or no suppression), whereas a shorter time to complete licks 76–100 of the pre-exposed mice compared to the non-pre-exposed mice. Data (i.e. suppression ratio) were analyzed using the Friedman nonparametric two-way ANOVA model. A dose of 2 mg/kg of amphetamine was administered ip, 30 min prior to preexposure and conditioning. SAR218645 and haloperidol were administered po, 60 min prior to pre-exposure and conditioning. Thirteen to fifteen animals per group were used.

#### Amphetamine-induced disruption of auditory evoked potentials (AEP) in rats

All recordings were made using Spike2 software (Cambridge Electronic Design, Cambridge, UK) running on a Windows PC connected to a 1401 interface (Cambridge Electronic Design, Cambridge, UK). EEG recordings were created by connecting the skull plug to an amplifying head stage (AI 1401, Axon Instruments, Union City, CA, USA), *via* a 6-channel commutator and cable (Plastics One, Roanoke, VA, USA). The signal was then further amplified with a multichannel amplifier (Cyberamp 380, Axon Instruments, Union City, CA, USA). Auditory stimuli were controlled by the interface *via* an eight channel power amplifier (SA8, Tucker Davis Technologies, Alachua, FL) connected to a patch panel (PP16, Tucker Davis Technologies), which was connected to speakers located above each cage (TDT Magnetic Speakers, Tucker Davis Technologies). During recording sessions, animals were placed inside standard mouse cages (30 × 15 × 15 cm) and rigged so that the six channel cables could connect with the rest of the apparatus outside of the cage.

##### Surgery

Male Sprague-Dawley rats (150–180 g at arrival) were anesthetized with 3% isoflurane and their scalp was shaved and prepared for surgery. An incision was made in the scalp and the skin was retracted to allow direct contact with the skull surface. The skull was cleaned with a dry Q-tip and five holes were drilled through the skull. Three holes were drilled at coordinates AP + 1 mm, ML + 1 mm (reference); AP−4 mm, ML ± 4 mm (leads 1 and 2) relative to bregma, while an additional hole was drilled in the frontal area (ground) and another hole was drilled at the midpoint between the anterior and posterior coordinate (anchor). Screw electrodes were then lowered through the skull so that they were in direct contact with the surface of the cortex. The leads from the screws were fed through a six channel pedestal (Plastics One, Roanoke, VA, USA) and the pedestal was secured to the skull surface with dental cement (Tylok Plus, Henry Schein, Melville, NY, USA). Once the cement had dried, the scalp was sutured and the animal was given an injection of Metacam (1 mg/kg).

AEP testing procedure: After approximately 4 weeks of recovery postsurgery, animals were tested on AEP gating over five sessions, each separated by 7 days, using a within subjects design wherein each animal was exposed to each drug treatment. The treatment conditions were vehicle + vehicle, vehicle + amphetamine (3 mg/kg), 0.3 mg/kg SAR218645 + amphetamine, 3 mg/kg SAR218645 + amphetamine, 0.3 mg/kg SAR218645 + vehicle and 3 mg/kg SSR180711 (reference α7 nicotinic receptor partial agonist) + amphetamine. Amphetamine was dissolved in 0.9% saline. Both SAR218645 and vehicle were administered po. All doses of saline and amphetamine were administered ip. For all gating sessions tone pairs consisted of two 1,500 Hz, 5 V tones, separated by a 500 ms interval, with a 10 s intertrial interval separating each double pulse. Sessions consisted of 350 tone pair presentations, each tone pair separated by 10 s, and lasted approximately 60 min. On each testing day, two sessions were administered. The first session occurred immediately after administration of vehicle or SAR218645 and consisted of 360 double pulse pairs (approximately 60 min). The second session occurred immediately after injection of vehicle or 3 mg/kg amphetamine and consisted of 360 double pulse pairs (again approximately 1 h). The second session followed immediately after the first.

AEP data analysis: Data were collected and analyzed using Spike2 software (CED Software, Cambridge, UK). Data were derived by smoothing raw waveforms and constructing a waveform average for each animal/channel. Then a horizontal cursor was placed at the highest and lowest (peak and trough) point of the P1/N1 wave, for both the wave occurring to the first tone (S1) and to the second tone (S2), and the value was recorded into an Excel spreadsheet. The P1 wave was defined as the highest peak occurring between 8 to 18 ms, and the N1 wave was defined as the lowest trough occurring between 18 and 35 ms. The amplitude of the S1 waveform was calculated by subtracting the value of the trough for the N1 wave from the value of the peak of the P1 wave. This was also done for the S2 waveform.

Then, a ratio was derived by dividing the value of the S2 wave from that of the S1 wave. Statistics performed on S2/S1 ratio consisted of using a one-way ANOVA, followed by a post-hoc Dunnett’s test for individual comparisons. Eleven to twelve animals per group were used.

## Methods: Investigation of potential side-effects

### Catalepsy in rats

Male Sprague-Dawley rats weighing 225–234 g were used. Each animal was placed such that their forepaws rested on a wooden dowel (1 × 18 cm) mounted horizontally 9 cm from the floor and 4 cm from one end of a white translucent plastic box (26 × 20 × 15 cm). The amount of time each rat spent with at least one forepaw on the bar was determined, for a maximum period of 540 s. This procedure was repeated three times. White noise was used during the acclimation and test periods to minimize the effects of outside noises. SAR218645 was administered alone at a dose of 30 mg/kg po. Haloperidol (1 mg/kg ip) was used alone and in co-administration with SAR218645 (1, 3, and 10 mg/kg, po). Statistics consisted of using a one-way ANOVA, followed by a post-hoc Dunnett’s test for individual comparisons. Ten animals per group were used.

### Effects of SAR218645 in models of seizures

#### The 6-Hz electroshock-induced seizures in mice

SAR218645 (10 and 30 mg/kg, po) and the reference anticonvulsant agent, levetiracetam (20 mg/kg), were administered ip. to OF1 mice (12–14 g) 30 min prior to test, respectively. An electrical stimulus (6 Hz, 0.2 ms rectangular pulse, 3 s duration, 32 mA) was applied to both corneas. Animals were closely observed and rated for seizure behavior according to a modified Racine scale^40^. Kruskal-Wallis multiple comparisons test was used to assess potential differences between treatment groups.

#### The maximal electroshock seizure (MES) test in mice

SAR218645 (30 mg/kg, po) and the reference anticonvulsant agent, diazepam (10 mg/kg), were administered ip to OF1 mice (12–14 g) 30 min prior to test, respectively. An electrical stimulus (60 Hz, 0.8 ms rectangular pulse, 0.4 s duration, 50 mA) was applied to both corneas. The occurrence of tonic extension of the hindlimbs was noted.

#### The pentylenetetrazol (PTZ) seizure threshold test in mice

The seizure threshold was determined by the infusion of 8 mg/mL of PTZ at a rate of 0.7 mL/min *via* a flexible plastic catheter into the tail vein of freely moving OF1 mice (22–24 g) using an infusion pump. The convulsant dose for each endpoint was calculated in mg/kg PTZ. SAR218645 (10 and 30 mg/kg, po) and diazepam (3 mg/kg, ip) were administered 60 or 30 min prior to the beginning of PTZ infusion, respectively. One-way ANOVA was used to assess overall effect of treatment, and Dunnett’s post-hoc was used to compare differences between treatment groups. Ten to fifteen animals per group were used.

## Results

### Synthesis of SAR218645

SAR218645 was synthesized in one step from compounds **3** and **6** as outlined in Fig. 1^28^. The preparation of intermediates **3** and **6** was realized following procedures reported respectively by Inoue *et al*.^26^ and Cao *et al*.^27^. The spectral data of SAR218645 and its precursors were consistent with the structure of SAR218645.

### Functional *in vitro* mGluR2 assays

#### Ca^2^+ mobilization assays

**Figure 2:**
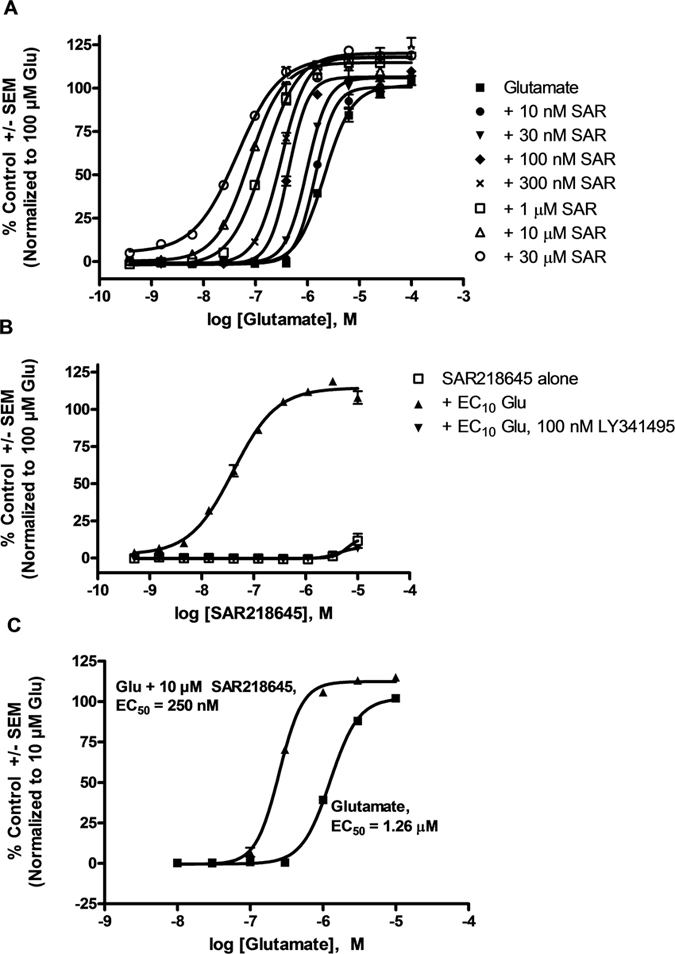
(**A,C**) Concentration-response curves for glutamate in the absence or in the presence of increasing concentrations of SAR218645 (SAR) in cell lines expressing rat mGluR2 and human mGluR2, respectively. (**B**) Concentration-response curve of SAR218645 in the presence of a submaximal concentration (EC_10_) of glutamate (Glu) in HEK293 cells stably expressing rat mGluR2 on Ca^2+^ mobilization. Antagonism of this effect by the mGluR2/3 receptor antagonist, LY341495. The fluorescence responses were normalized as a percentage of the maximal response to glutamate (10 or 100 μM) and represent the means ± S.E.M.

Activation of mGluR2 was assessed by measuring glutamate-induced increases in intracellular calcium in HEK293 cells expressing rat mGluR2 coexpressed with the G protein (Gα16). Consistent with the effects of other allosteric potentiators of mGluR2, increasing concentrations of SAR218645 induced parallel leftward shifts of the glutamate concentration-response curves in the rat mGluR2 HEK293 cells with no significant increase in the maximal response to glutamate (Fig. 2A). SAR218645 enhanced glutamate potency of approximately 25-fold (Fig. 2A). To assess the potency of SAR218645, we determined the effects of increasing concentrations of SAR218645 on the response to an EC_10_ concentration (100 nM) of glutamate in HEK293 cells expressing rat mGluR2/Gα16 (Fig. 2B). SAR218645, while having no effect *per se*, enhanced sensitivity to glutamate, an effect abolished by the mGluR2/3 antagonist, LY341495 (Fig. 2B). Similar to the potentiation observed in rat mGluR2, SAR218645 also produced a concentration-dependent potentiation of the glutamate response of HEK293 cells expressing human mGluR2/Gα16 with a maximal potentiation of approximately 10-fold and an EC_50_ value of 250 nM (Fig. 2C).

**Figure 3:**
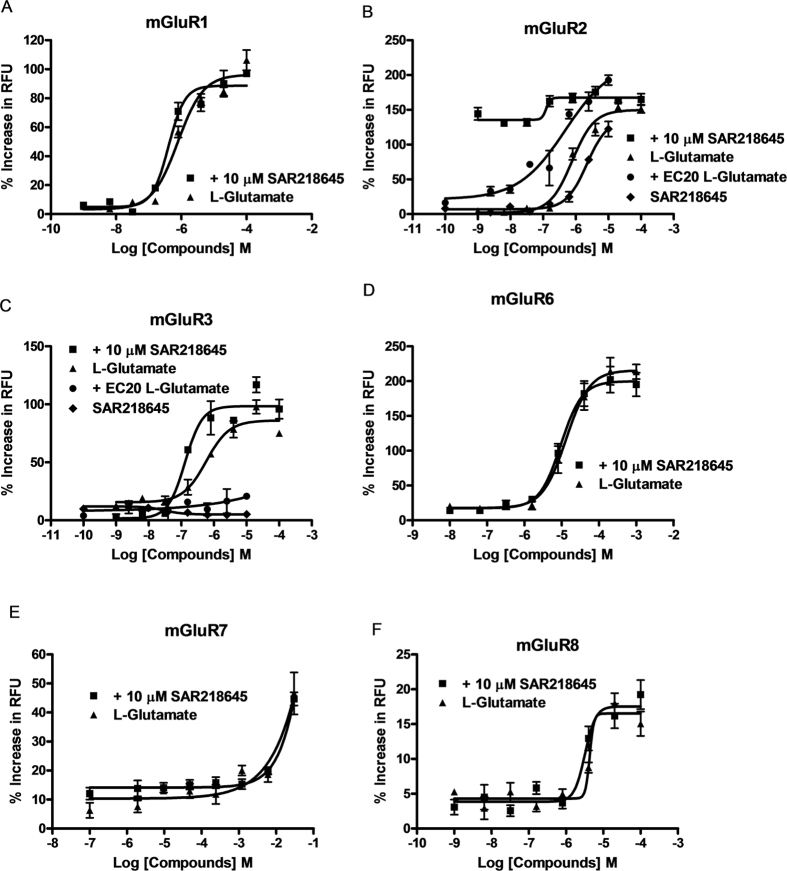
Concentration-dependent intracellular calcium increases stimulated by the agonist L-glutamate in the presence and in the absence of 10 μM SAR218645 was measured in cells expressing mGluRl, mGluR2, mGluR3, mGluR6, mGluR7 and mGluR8. Concentration-dependent intracellular calcium increases stimulated by SAR218645 in the presence or in the absence of EC_20_ of agonist L-glutamate was measured in cells expressing mGluR2 and mGluR3.

To determine the selectivity of SAR218645 for mGluR2 relative to that of other mGluR receptor subtypes, the effects of this compound on glutamate-induced increases in intracellular calcium in HEK293 cells expressing human mGluR1, human mGluR3, human mGluR6, human mGluR7 and human mGluR8 coexpressed with the G protein, Gqi5, was assessed. In particular, the ability of SAR218645 to shift glutamate-induced increases in intracellular calcium in cells expressing human mGluR3 was measured (Fig. 3C). A control experiment using this cell preparation confirmed that increasing concentrations of SAR218645 produced a leftward shift in the glutamate response in cells expressing human mGluR2 (Fig. 3B). In contrast, SAR218645 had no effect on glutamate-induced increases in intracellular calcium in human mGluR3 (Fig. 3C). Moreover, it had no effect on glutamate-induced activation of the other mGluR subtypes tested (Fig. 3A,D–F). Furthermore, application of SAR218645 alone did not elicit a calcium response in any of the other cell lines tested (Fig. 3A,C–F). These data suggest that SAR218645 is a potent and highly selective positive allosteric modulator of mGluR2.

#### Measurement of cAMP accumulation

**Figure 4:**
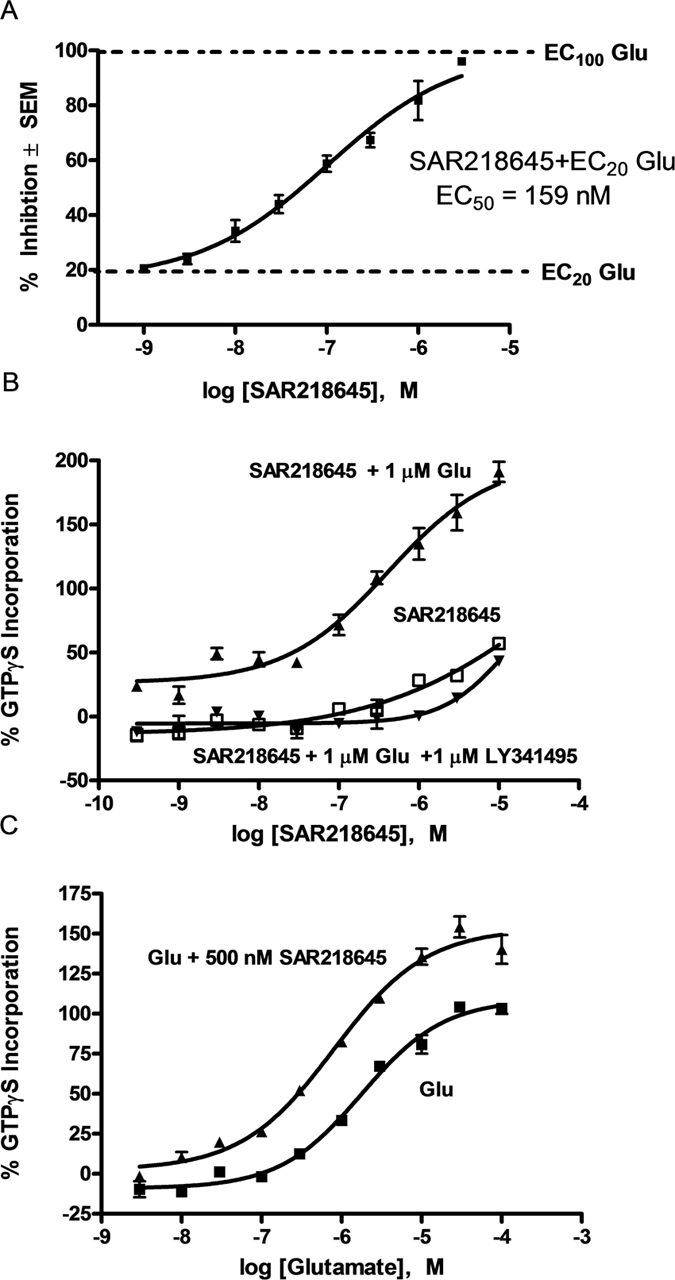
(**A**) SAR218645 potentiation of glutamate (Glu) inhibition on 1 μM forskolin-stimulated cAMP production in HEK293 cells expressing human mGluR2. The assay was performed in the presence of an EC_20_ concentration of glutamate. Data represent the mean ± S.E.M. (**B**,**C**) [^35^S]GTPγS binding to mouse cortex. (**B**) Concentration-effect curve for SAR218645 on the binding of [^35^S]GTPγS in the presence or in the absence of glutamate (Glu). Antagonism of this effect by the mGluR2/3 receptor antagonist, LY341495. (**C**) Stimulation of [^35^S]GTPγS binding produced by glutamate (Glu) in the absence or in the presence of SAR218645. Data are expressed as percentage of the maximal response to glutamate (100 μM) and are mean ± S.E.M.

Glutamate inhibited forskolin-stimulated cAMP accumulation in HEK293 cells expressing human mGluR2 in a concentration-dependent manner. SAR218645 enhanced the inhibitory effect of glutamate at an EC_20_ value concentration of 100 μM on forskolin-stimulated cAMP accumulation in a concentration-dependent manner, with an EC_50_ value of 159 nM (Fig. 4A).

#### [^35^S]GTPγS binding to mouse cortex

SAR218645 potentiates a glutamate-stimulated increase in [^35^S]GTPγS at an equivalent EC_20_ value (1 μM), shifting the glutamate concentration-effect curve both upward and leftward resulting in an EC_50_ value for glutamate of 415 nM (Fig. 4B). This effect was blocked by the mGluR2/3 antagonist, LY341495. It is noteworthy that SAR218645 acted as a pure mGluR2 PAM at native mouse mGluR2 in the cortex (i.e., did not have any intrinsic activity in the absence of glutamate). Furthermore, SAR218645 (500 nM) produced a leftward shift in the glutamate-induced [^35^S]GTPγS binding, resulting in an EC_50_ value for glutamate of 802 nM (Fig. 4C).

#### [^3^H]LY341495 binding

**Figure 5:**
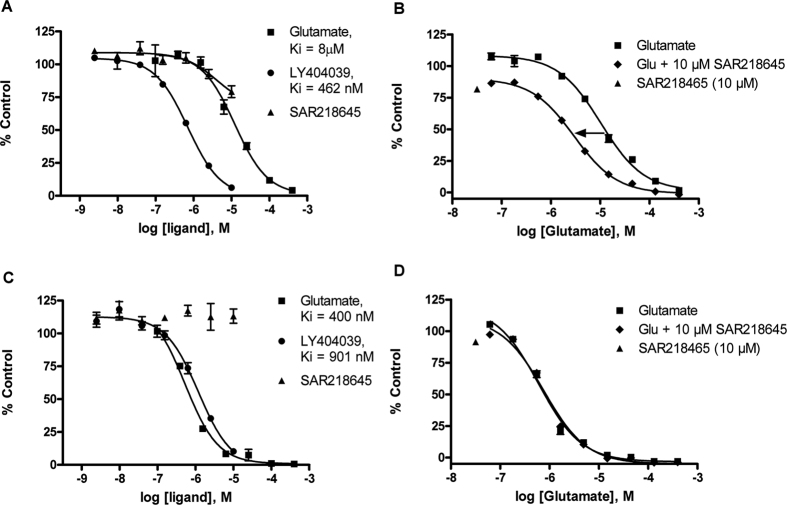
Displacement of [^3^H]-LY341495 binding to rat recombinant mGluR2 (**A,B**) or mGluR3 (**C,D**) by SAR218645, glutamate (Glu) or the mGluR2/3 agonist, LY404039 alone, or in combination (SAR218645 and glutamate). Data are expressed as percentage of specific binding ± S.E.M.

To confirm the allosteric character of SAR218645, the drug was tested for binding displacement of the prototypic orthosteric (i.e., glutamate-site) mGlu2/3 receptor antagonist, [^3^H]LY341495, to rat mGluR2-expressing membranes. [^3^H]LY341495 binding to rat mGluR2 was not inhibited upon the addition of 10 μM SAR218645 (Fig. 5A). In contrast to the lack of effect of SAR218645 on the binding of the antagonist radioligand, increasing concentration of glutamate and the orthosteric mGluR2/3 agonist, LY404039, inhibited [^3^H]LY341495 binding (Fig. 5A). Glutamate-mediated inhibition of [^3^H]LY341495 binding was assessed in the absence and presence of 10 μM SAR218645. Results showed that SAR218645 not only decreased the maximal binding (108 vs 90%) but also increased the affinity of glutamate, i.e., lowered the Ki of glutamate by 3.5-fold (6.8 vs 1.9 μM) (Fig. 5B). To confirm the selectivity of SAR218645 for mGluR2 relative to the mGluR3 receptor subtype, the drug was tested for displacement of [^3^H]LY341495 binding to rat mGluR3-expressing membranes. Results showed that SAR218645 had no effect on [^3^H]LY341495 binding to the mGluR3 when applied alone (Fig. 5C), nor did the drug affect glutamate-mediated inhibition of [^3^H]LY341495 binding on membranes expressing mGluR3 (Fig. 5D).

#### *In vitro* selectivity profile

When SAR218645 was tested at a concentration up to 10 μM for inhibition of radioligand binding to a battery of neurotransmitter and peptide receptors, ion channels, transporters and kinases, results showed no interaction with any of these targets (data not shown, see Table S1 for the complete list).

#### Plasma exposure and brain penetration

Single oral administration of SAR218645 at 1, 3 and 10 mg/kg in mice resulted in a near dose-proportional increase in compound concentration in mouse plasma and brain. Peak brain concentrations (Cmax) were observed 1 h after SAR218645 administration (Table 1).

### Functional *in vivo* assays

#### LY404039-induced turning behavior in mice

**Figure 6:**
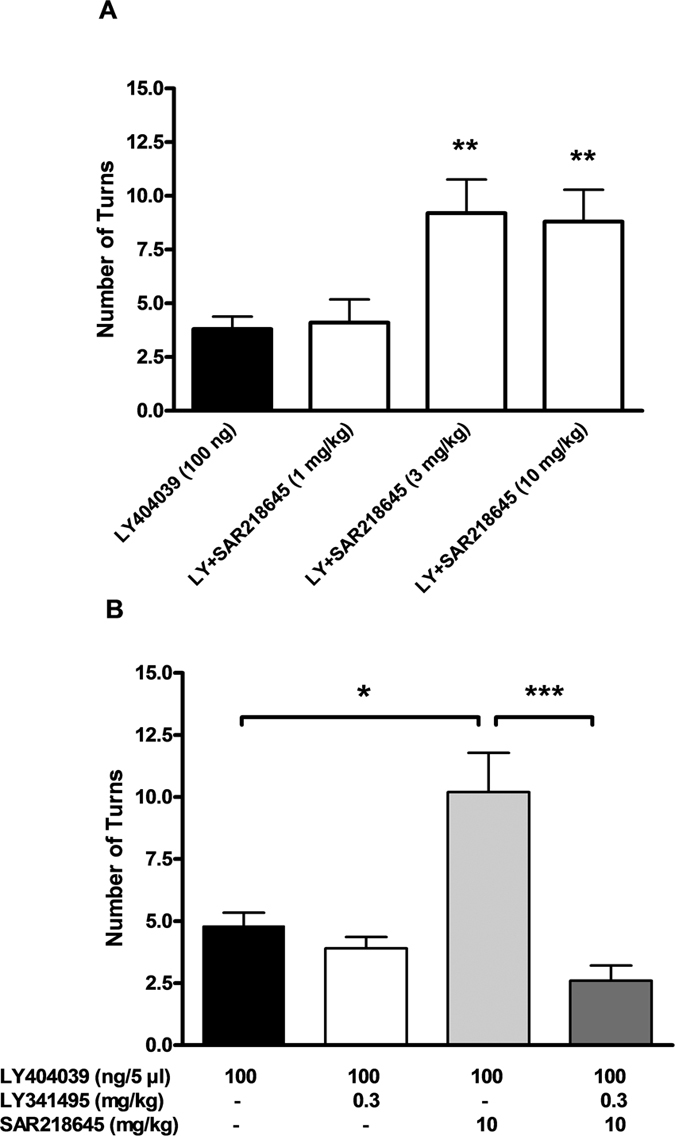
SAR218645 potentiation of mouse turning behavior induced by unilateral intracortical administration of the mGluR2/3 agonist, LY404039. (**A**) Dose-response; (**B**) antagonism of the effects of SAR218645 given orally by the mGluR2/3 antagonist, LY341495. Data represent Mean + S.E.M. *P < 0.05, **P < 0.01 and ***P < 0.001 vs. LY404039 (Wilcoxon two-tailed comparison vs. LY404039). N = 10 per group.

LY404039-induced turning behavior was potentiated significantly by the peripheral administration of SAR218645 at 3 and 10 mg/kg (χ^2^ = 14.69, P < 0.01) (Fig. 6A). This effect of SAR218645 (10 mg/kg) was blocked by the mGluR2/3 antagonist, LY341495, administered at 0.3 mg/kg (χ^2^ = 20.76, P < 0.001) (Fig. 6B).

### Assessment of mGluR2-5-HT_2A_ receptor interaction

#### DOI-induced glutamate release in the rat medial prefrontal cortex

**Figure 7:**
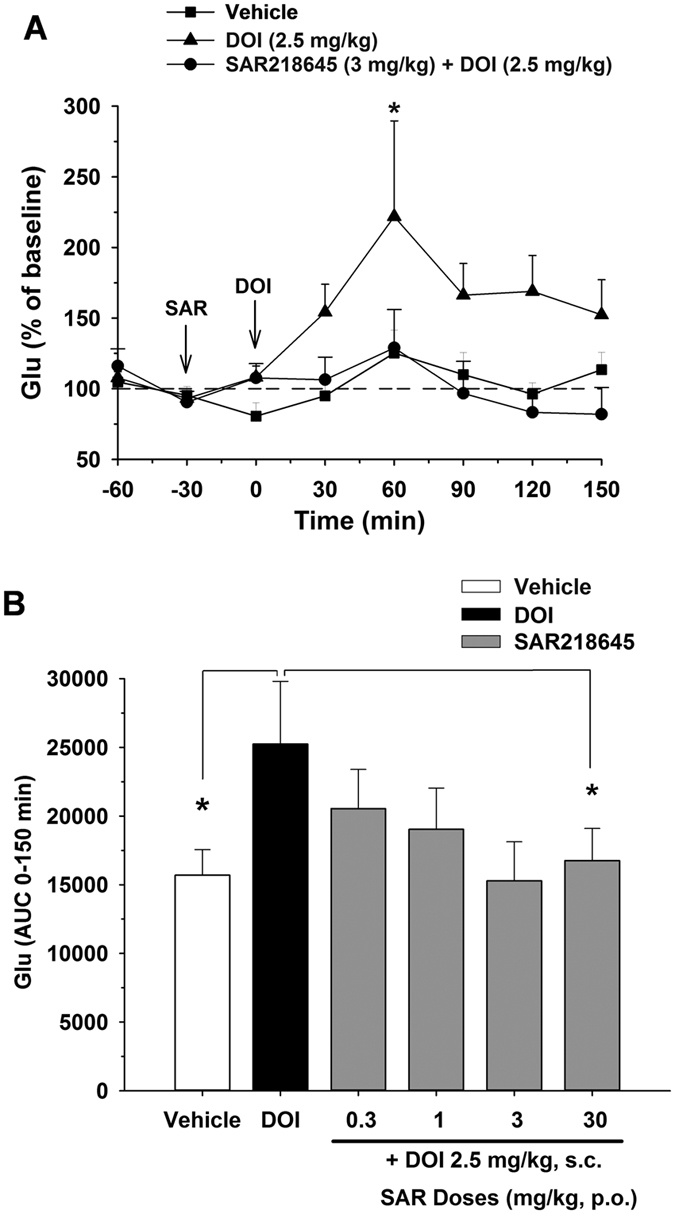
Effect of SAR218645 on DOI-induced glutamate (Glu) release in the medial prefrontal cortex measured by microdialysis in freely-moving rats. Data are expressed as (**A**) percent of basal level over 150 min or (**B**) AUC, and represent Mean + S.E.M. *P < 0.05 vs. DOI, Dunnett’s test (Panels A and B, SAR218645 versus DOI-treated group) or Student’s t-test (Panel B, vehicle versus DOI). N = 5 to 9 per group.

Subcutaneous administration of the 5-HT_2A_ receptor agonist, DOI at 2.5 mg/kg, produced a rapid increase in the extracellular levels of glutamate in the prefrontal cortex of freely moving rats (Fig. 7A). This effect was blocked significantly by pretreatment with SAR218645 at 3 mg/kg (Fig. 7A). Areas under the curve during the 150 min after drug injection confirm the effect of SAR218645 at 3 and 30 mg/kg, with this latter dose reaching statistical significance (P < 0.05) (Fig. 7B).

## Results: Effects of SAR218645 in models of schizophrenia-related symptoms

### Models predictive of therapeutic activity against positive symptoms

#### DOI-induced head-twitch behaviors in mice

**Figure 8:**
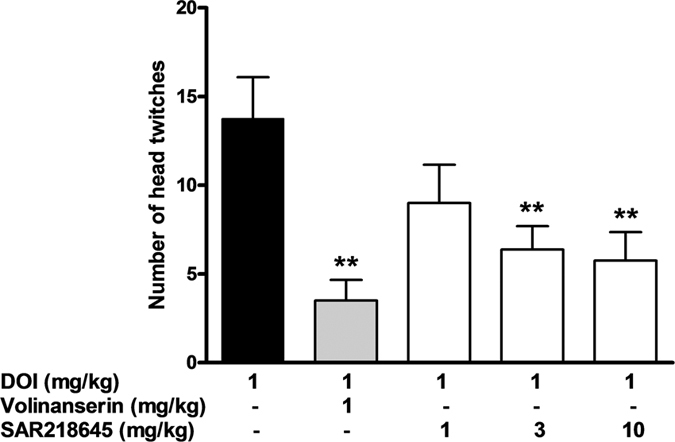
Antagonism by SAR218645 of DOI-induced head-twitch behavior. Comparison with the 5-HT_2A_ receptor antagonist, volinanserin. Data represent Mean + S.E.M. (dose-response). **P < 0.01 (Newman-Keuls test). N = 8 per group.

Like the 5-HT_2A_ receptor antagonist, volinanserin, SAR218645 dose-dependently inhibited head twitches induced by the hallucinogen, DOI [F(4, 35) = 6.16, P < 0.001]. Post-hoc analysis showed that this effect reached statistical significance at 3 and 10 mg/kg (Fig. 8).

#### Locomotor hyperactivity in wild-type and transgenic mice

**Figure 9:**
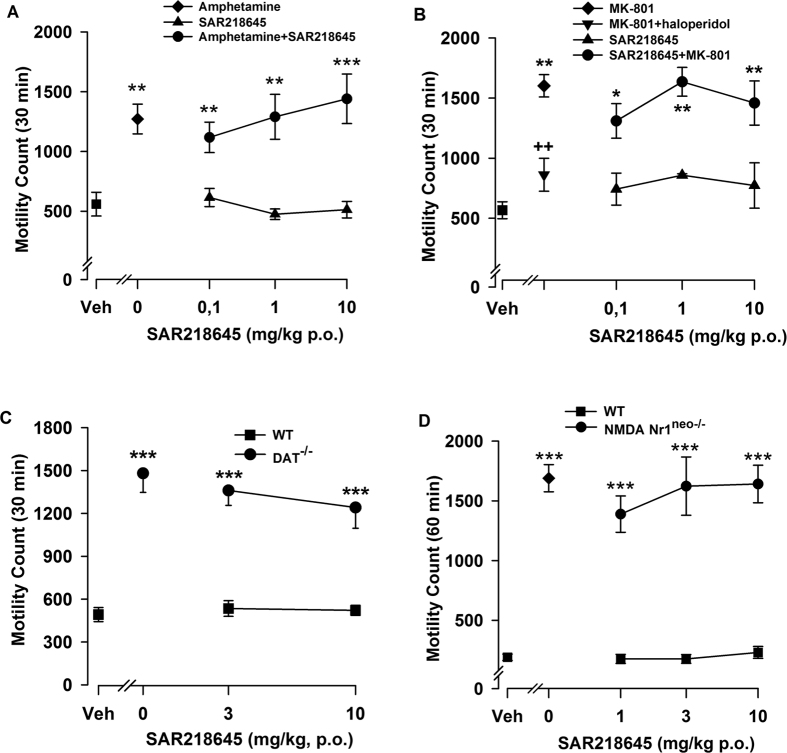
Effects of SAR218645 on motor hyperactivity in male Swiss mice induced by (**A**) amphetamine (2 mg/kg, ip) or (**B**) MK-801 (0.2 mg/kg, ip), or on spontaneous locomotor hyperactivity of (**C**) DAT^−/−^ or (**D**) Nr1^neo−/−^ transgenic mice. Each symbol represents the mean (±S.E.M.) number of beam breaks recorded for 30 or 60 min, 60 min following the po administration of SAR218645, haloperidol (0.3 mg/kg) or vehicle. Post hoc analysis following one-way ANOVAs and Dunnett’s tests: **P < 0.01, ***P < 0.001 as compared with the absolute control (WT/vehicle) for the hyperactive group (i.e, amphetamine, MK-801, DAT^−/−^ or Nr1^neo−/−^/vehicle) in each experiment. ^++^P < 0.01 for significant decreasing effects of the tested compound on the hyperactivity as compared with the hyperactive considered group (MK-801/vehicle). N = 5 to 9 per group.

There were significant main effects of treatment or genotype on locomotor activity [MK-801: F(8, 63) = 7.53, P < 0.001; Amphetamine: F(7, 64) = 10, P < 001; NMDA Nr1^neo−/−^: F(7, 35) = 37.21, P < 0.001; and DAT^−/−^: F(4, 49) = 13.58, P < 0.001], but in each case there were no effects of SAR218645 treatment (see Fig. 9).

#### Conditioned Avoidance Response (CAR) in mice

One-way ANOVA revealed a significant main effect of treatment [F(4, 35) = 20.32, P < 0.001]. Post-hoc comparisons showed that olanzapine, but not SAR218645, significantly decreased avoidance responses (Table 2).

### Models predictive of therapeutic activity against the cognitive symptoms

#### The novel object recognition task in mice

**Figure 10:**
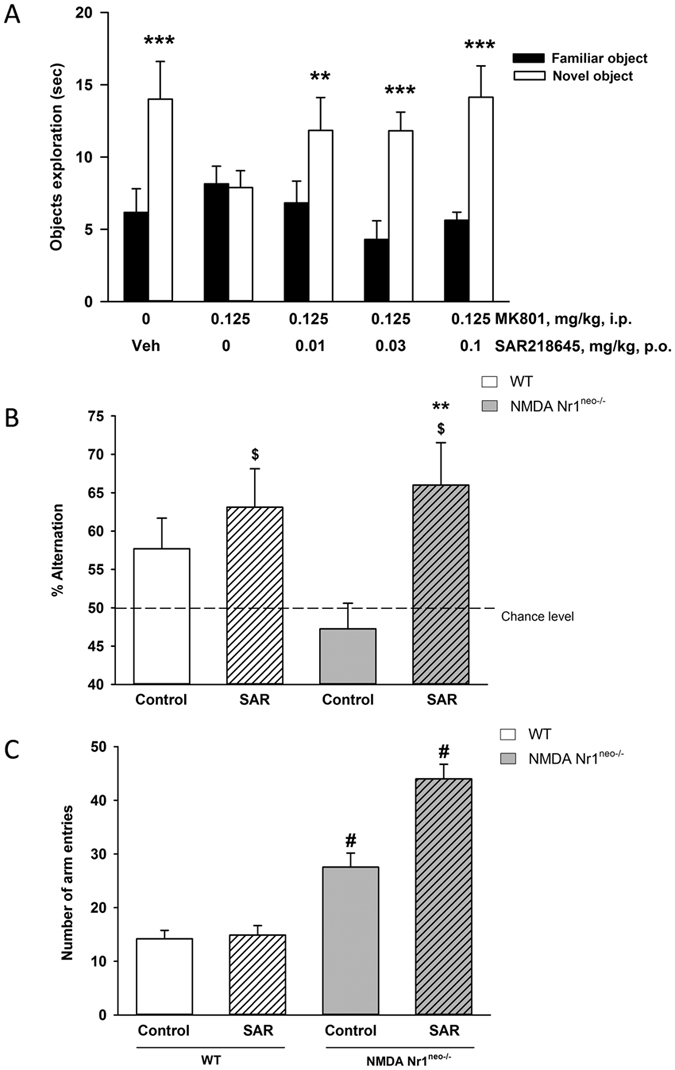
(**A**) Reversal by SAR218645 of an MK-801-induced deficit of episodic memory in an object recognition task in mice. Each bar represents the average (+SEM) time spent exploring a novel or a familiar object. The delay between the acquisition and the recall session was 60 min. Post hoc analyses following a two-way ANOVA: **P < 0.01 and ***P < 0.001 novel vs familiar object at the considered treatment condition. N = 8 to10 mice per group. (**B**,**C**) Effect of a single administration of SAR218645 (0.1 mg/kg, po, SAR) on (**B**) spontaneous alternation and (**C**) number of arm entries in the Ymaze test in wild-type (WT) and NMDA Nr1^neo−/−^ mice. Bars represent Mean + S.E.M. **P < 0.01 (vs. control), ^$^P < 0.05 (vs. chance level) and ^#^P < 0.001 (vs. WT). N = 10–11 mice per group.

Control mice spent a greater amount of time investigating the novel object (14.01 vs 6.17 s). This preferential investigation of the novel object was abolished (7.89 vs 8.15 s) by administration of MK-801 (0.0125 mg/kg, ip) immediately after presentation of the familiar object. SAR218645, at 0.01, 0.03 and 0.1 mg/kg po, when coadministered with MK-801, significantly restored this preferential investigation (Winer analysis comparing the time spent investigating the novel vs the familiar object, following significant treatment x object interaction effects [two-way ANOVA: F(3, 33) = 7.64, P < 0.001] (Fig. 10A).

#### The Y-maze test in NMDA Nr1^neo−/−^ mice

Transgenic animals treated with saline displayed fewer spontaneous alternations than their WT counterparts, but 2-way ANOVA failed to reached statistical significance (F1, 37 = 2.19, P = 0.15) (Fig. 10B). Moreover, analysis using *t*-test indicated that the performance of both control groups did not differ significantly from the chance level (WT: t = 1.93; NMDA Nr1^neo−/−^: t = −0.82), while those from the two groups treated with SAR218645 (SAR; 0.1 mg/kg, po) were significantly different from the chance level (WT/SAR: t = 2.60, P < 0.05; NMDA Nr1^neo−/−^/SAR: t = 2.89, P < 0.05). Transgenic animals treated with SAR218645 displayed significantly more spontaneous alternations than those treated with saline [F(1, 37) = 7.19, P < 0.05] (Fig. 10B). Finally, both transgenic groups displayed increased arm entries when compared to their WT counterparts [2-way ANOVA; F(1, 37) = 12.24, P < 0.001] (Fig. 10C).

#### Amphetamine-induced disrupted latent inhibition (LI) in mice

**Figure 11:**
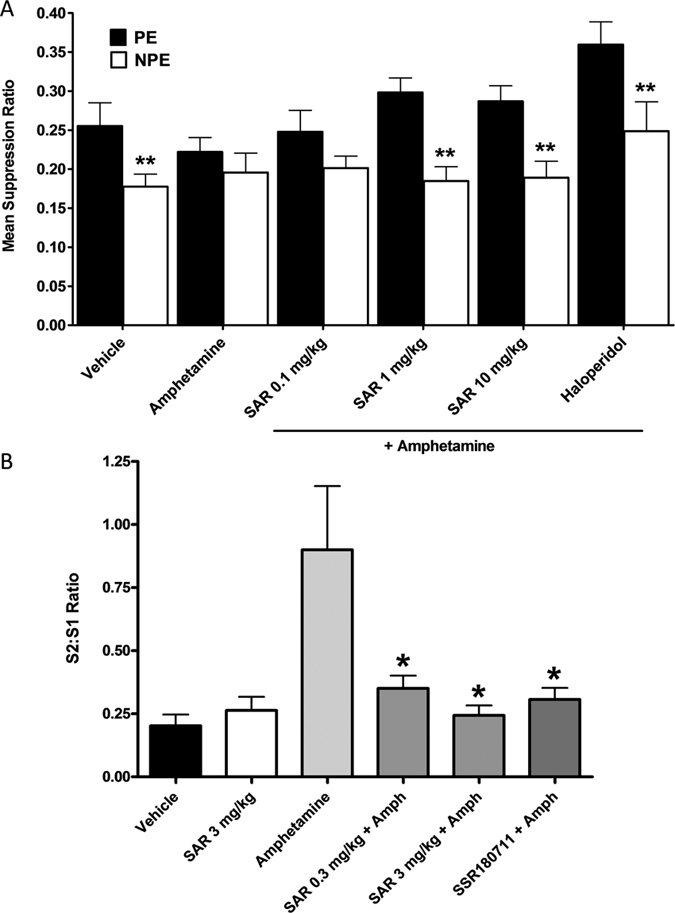
(**A**) Effects of SAR218645 on amphetamine-induced LI disruption. Data represent mean (+SEM) suppression ratio after tone onset of the pre-exposed (PE; white bars) and non pre-exposed (NPE; black bars) mice treated with amphetamine at 2 mg/kg ip or vehicle, and pretreated with either SAR218645 at doses of 0.1, 1, or 10 mg/kg po, haloperidol at 0.3 mg/kg po or vehicle. Asterisks indicate a significant difference between the PE and NPE groups, namely, presence of LI (**P < 0.01). N = 13 to 15 per group. (**B**) Effects of SAR218645 and the α7 nicotinic receptor partial agonist, SSR180711 (3 mg/kg), on amphetamine-induced disruption of auditory evoked potentials in the rat. S1, wave occurring to the first tone, and S2, wave occurring to the second tone. Bars represent Mean + S.E.M. *P < 0.05 (versus amphetamine alone). N = 11 to 12 per group.

The experimental groups did not differ in their times to complete licks 51–75 before tone onset. Mice treated with vehicle exhibited LI whereas LI was absent in mice treated with amphetamine (Fig. 11A). Two-way ANOVA yielded significant main effects of pre-exposure (P < 0.0001) and treatment (P < 0.05). Post-hoc comparisons revealed a significant difference between the pre-exposed and non pre-exposed groups in the vehicle-vehicle, amphetamine-SAR218645 at 1 and 10 mg/kg, and amphetamine-haloperidol conditions (Fig. 11A).

#### Amphetamine-induced disruption of auditory evoked potentials in the rat

In this study, there was a significant effect of treatment on auditory gating as measured by the S2/S1 wave amplitude ratio [F(4, 44) = 5; P < 0.01]. Amphetamine (3 mg/kg) significantly disrupted auditory evoked potential gating. SAR218645 (3 mg/kg, po) did not affect baseline gating, but it attenuated amphetamine-induced disrupted gating at doses of 0.3 and 3 mg/kg, as did the reference α7 nicotinic receptor agonist, SSR180711, at 3 mg/kg po (Fig. 11B).

## Results: Investigation of potential side-effects

### Catalepsy in rats

One-way ANOVA revealed a significant main effect of treatment [F(4, 45) = 36, 25, P < 0.001]. Post-hoc analysis showed that haloperidol at 1 mg/kg, but not SAR218645 at 30 mg/kg, induced catalepsy. When SAR218645 at 3 or 30 mg/kg was co-administered with haloperidol at 1 mg/kg, it did not affect haloperidol-induced catalepsy (Table 3).

### Effects of SAR218645 in models of seizures

#### The 6-Hz- and maximal-electroshock-induced seizures, and pentylenetetrazol (PTZ) seizure threshold tests in mice

SAR218645 was devoid of any activity on seizures induced by 6-Hz and maximal stimulation, and by the convulsant PTZ (Table 4). This was in contrast to the reference anticonvulsant, levetiracetam and the benzodiazepine, diazepam, which afforded full protection in the 6-Hz [F(3, 60) = 11.89, P < 0.001], maximal and PTZ [F(3, 52) = 159.78, P < 0.001] tests, respectively (Table 4).

## Discussion

Enhancing glutamate signaling in the forebrain is a potential strategy to treat schizophrenia. Initially, orthosteric mGluR2/3 agonists were developed as potential treatment candidates, but these compounds failed to demonstrate convincing clinical efficacy in this condition, and additionally, some of them were found to produce centrally-mediated side effects, which seriously limits their use on a routinely basis^14^. However, new generations of glutamate-enhancing compounds that allosterically enhance the functionality of mGluR2 have been discovered. These novel drugs are thought to be more selective and safer than direct agonists based on the assumption that they are active only at sites of on-going production of glutamate. Nonetheless, despite sometimes compelling preclinical data^7^, the road from preclinical idea to successful clinical trial has also been challenging. Indeed, recently an mGluR2 PAM (AZD8529) failed in clinical trials for schizophrenia^41^. Here we describe the *in vitro* and *in vivo* profile (Table 5) of SAR218645, a highly selective, brain penetrant and orally-active mGluR2 PAM. In addition, we suggest modifications to the clinical development of molecules such as SAR218645, that could increase the possibility of success in human.

### Characterization of the mechanism of action of SAR218645

Allosteric modulators can enhance the efficacy of the endogenous agonist by modifying its affinity^42^. Here, SAR218645 was able to increase the affinity of glutamate, with the Ki for [^3^H]LY341495 binding to recombinant rat mGluR2 decreasing by 3.5-fold. As expected, SAR218645 did not alter the binding of the orthosteric antagonist when applied alone. Moreover, [^3^H]LY341495 binding to recombinant rat mGluR3 was not altered by SAR218645, either alone, or in combination with glutamate. Further, *in vitro* studies showed that SAR218645 potentiated glutamate-stimulated [^35^S]GTPγS binding to mouse native mGluR2. In intracellular assays, SAR218645, while inactive on its own, facilitated the Ca^2+^ response to glutamate in cells expressing mGluR2, but not in cells expressing any of the other mGluR subtypes. The PAM activity of SAR218645 at mGluR2 was further demonstrated in another cell-based assay, where the drug enhanced the inhibitory effect of glutamate on forskolin-stimulated cAMP formation. Finally, these findings were confirmed in a behavioral experiment showing that SAR218645 was able to potentiate the effects of the orthosteric mGluR2/3 agonist, LY404039. Together, these results indicate that SAR218645 acts as an mGluR2 PAM displaying functional selectivity for mGluR2 relative to any of the other mGluR subtypes.

### Characterization of SAR218645 in models of psychosis

SAR218645 was tested in several experimental procedures claimed to model certain aspects of the positive symptoms of schizophrenia. Results showed that while the drug failed to inhibit conditioned avoidance and hyperactivity in several pharmacological and transgenic models, it reduced head twitch behavior in mice induced by the 5-HT_2A_ receptor agonist, DOI. These behavioral findings agree with previous studies showing similar effects of mGluR2 ligands against DOI-induced behavioral responses. For example, Gewirtz and Marek^43^ were the first to report that activation of mGluR2/3 receptors by the selective agonist LY354740, suppressed DOI-induced head twitch behavior in rats and these findings have been replicated several times by a number of different research groups using both orthosteric agonists and PAMs of the mGluR2 (for a recent review, see ref. 44).

Several studies have shown the production of positive symptoms, such as delusions, by hallucinogenic drugs that are known to function as 5-HT_2A_ receptor agonists^45^. The 5-HT_2A_ receptor is believed to be involved in the mechanism of action of atypical antipsychotics^46^,^47^. Moreover, the 5-HT_2A_ receptor and mGluR2 form a specific heterodimeric GPCR complex in heterologous expression systems, which is distributed throughout the mouse and human frontal cortex^48^. Extensive electrophysiological, biochemical and behavioral experiments demonstrate an antagonistic interaction between 5-HT_2A_ receptors and mGluR2 at the level of the prefrontal cortex, with activation of mGluR2 reducing 5-HT_2A_ receptor-induced responses^44^. The current findings that SAR218645 not only blocked the behavioral response of DOI, but also decreased glutamate release in the prefrontal cortex following 5-HT_2A_ receptor activation fit well with these observations, and strengthen further the idea that the prefrontal cortex may play a crucial role in the action of mGluR2 PAMs.

As indicated above, SAR218645 did not attenuate hyperactivity involving either drug challenge (i.e., amphetamine and MK-801) or in transgenic mice (i.e., NMDA Nr1^neo−/−^ and DAT^−/−^). While the lack of effect of SAR218645 against amphetamine agrees with previous studies^19^,^21^, the inability of SAR218645 to affect hyperlocomotion induced by the NMDA receptor antagonist, MK-801, contradicts results from previous studies in mice showing that mGluR2 PAMs were able to block hyperactivity induced by the NMDA receptor antagonist, PCP^15^,^19^,^20^,^21^. MK-801 has been designated a PCP-type drug, sharing many effects of PCP, including impact on learning and motor activity, effects claimed to involve NMDA receptor blockade^49^. However, additional mechanisms by which MK-801 alters behavior, for example a potentiation of serotonergic function involving 5-HT_1A_ receptors, have been proposed^50^,^51^. Whether this may explain the difference in profile between SAR218645 vs. MK-801 and previous mGluR2 PAMs against PCP remains to be clarified. It is important to note that the inability of SAR218645 to affect the behavior in all four hyperactivity models is not due to poor pharmacokinetic (PK) properties of the drug, in particular with respect to brain exposure as our PK study showed that the drug readily enters into the brain and that brain concentrations at the doses tested in these models were sufficient to exert a pharmacological activity as demonstrated in the DOI-induced head-twitch study. Taken altogether, the findings with SAR218645 in models of psychosis, do not convincingly support the idea that this mGluR2 PAM may have a potential in treating the positive symptoms of schizophrenia. While the effects of SAR218645 against the hallucinogenic drug DOI may suggest that allosteric activation of mGluR2 may represent a potential strategy to attenuate hallucinogenic symptoms in schizophrenia, it is not clear how such a specific action may translate into the clinic and the treatment of a heterogeneous condition such as schizophrenia. Since positive symptoms of schizophrenia can cause severe debilitation in patients, we would not expect SAR218645 to be successful as a monotherapy for schizophrenia.

### Characterization of SAR218645 in translational models of cognitive impairment associated with schizophrenia

The cognitive deficits observed in schizophrenic patients are considered to form a core set of symptoms of this condition, representing a major hurdle to reinsertion and proper societal functioning of patients^52^,^53^. These deficits include attentional disturbances, memory impairment (more particularly working, episodic, and sequential memory), and information processing deficits. In the present study, SAR218645 was active in several models addressing certain aspects of cognitive impairment and attention deficits associated with schizophrenia.

#### Effects of SAR218645 in models of learning and memory

Our results demonstrate that SAR218645 ameliorated NMDA receptor blockade/deficit-induced impairment of several types of memory. SAR218645 improved memory performance in the object recognition task, using a short-term episodic memory protocol with animals impaired by an acute dose of MK-801. It is important to note that SAR218645 attenuated the deleterious effects of MK-801 when the psychotomimetic was administered in the immediate post-acquisition interval, thus affecting consolidation and/or recall processes. Moreover, SAR218645 attenuated a working memory deficit in the Y-maze test in NMDA Nr1^neo−/−^ mice, which express low levels (i.e., 5–10%) of functional NMDA receptors^33^. This is the first experimental evidence that an mGluR2 PAM is able to ameliorate learning impairment in models of NMDA receptor dysfunction. The literature on the effects of orthosteric mGluR2/3 agonists in models of learning and memory is inconsistent, with the seminal study by Moghaddam and Adams^4^ showing that LY354740 attenuated the disruptive effects of PCP on working memory, while later experiments with the same or different mGluR2/3 agonists failed to replicate this finding or even showed that they can impair memory function under normal conditions (i.e. in the absence of NMDA receptor blockade)^54^,^55^,^56^,^57^,^58^,^59^. The reason for this inconsistency in drug effect remains to be elucidated, but these findings argue against the therapeutic use of orthosteric agonists, which may produce unwanted effects under certain circumstances.

#### Characterization of SAR218645 in translational models that could be used to define clinical development

SAR218645 was tested in LI, a cross-species selective attention phenomenon that is disrupted in patients with schizophrenia and rodents treated with psychostimulants^60^. Amphetamine at low doses has been shown to abolish LI by favoring switching to respond according to the stimulus-reinforcement contingency and processing the irrelevant stimulus as if it were novel, thus impairing the capacity to in-attend to irrelevant stimuli. Here, SAR218645 restored the capacity to ignore irrelevant stimuli in amphetamine-treated rats and enabled flexible re-deployment of attentional resources according to current situational demands.

LI is a learning process observed when the acquisition of a conditional response to a conditioned stimulus paired with a reinforcer is retarded if the same stimulus has previously been pre-exposed in the absence of the reinforcer. It has been suggested that because certain symptoms of schizophrenia are characterized by an inability to filter, or ignore irrelevant stimuli, that LI is an endophenotype of schizophrenia^61^,^62^. However there are many contradictory results regarding the abnormality (or lack of) in schizophrenia^63^. There appears to be a difference between positive and negative symptoms in relation to the expression of LI, with preponderance for high levels of negative symptoms. Nonetheless, LI could potentially be used as a screen for schizophrenic patients in a clinical trial were LI improvement was seen in the preclinical setting. We suggest that the failure of predecessor molecules was potentially due to patient selection, e.g., broad patients recruitment criteria. Here we have a molecule effective in reversing LI abnormalities, perhaps those patients with the most abnormal LI may be best suited to a molecule such as SAR218645.

Moreover, SAR218645 was tested against amphetamine-induced alteration in AEP peak amplitude. Schizophrenia patients display abnormalities in auditory event-related potentials including an alteration in P50 suppression by reduced amplitude of the first stimulus tone (S1), which are assumed to reflect deficits in attention and processing speed assessed in cognitive tasks^64^,^65^,^66^,^67^,^68^,^69^. In this study, amphetamine disrupted the S2/S1 ratio for P1 and N1 peaks, reflecting respectively pre-attentive and attentional processing, due to reduced suppression of the response to the first stimuli, which is in line with previous reports showing a consistent reduction in both the amplitude to the first stimuli and the gating index^70^,^71^,^72^. SAR218645 restored the deficit in pre-attentive stages of sensory information processing induced by amphetamine, an effect shared by the α7-nACh receptor partial agonist, SSR180711, which was previously shown to improve impaired cognitive and attentional processes in a variety of experimental procedures^38^,^73^. The literature on the effects of mGluR2 signaling modulation on disrupted selective attention and sensory gating is sparse. An earlier study using the mGluR2 PAM, LY487379, demonstrated that the drug ameliorated cognitive flexibility in an attentional set-shifting task^74^. More recently, Ahnaou and colleagues^70^ showed that the orthosteric mGluR2 agonist, LY404039, is able to attenuate the disruptive effects of amphetamine and PCP on auditory evoked oscillations and potentials.

In a similar scenario to that of LI, P50 gating is a well recognized endophenotype of schizophrenia^75^,^76^,^77^. There is a large body of evidence to suggest that many (but not all) patients with schizophrenia have P50 gating abnormalities^78^. Following on from the proposals made in the LI section. One could envision a clinical trial for SAR218645 where only those patients with severe P50 suppression would be enrolled. The hypothesis would be that those patients with little or no P50 suppression would not benefit from a molecule such as SAR218645. As SAR218645 reverses suppressed P50 gating we may be able to select those patients who would see the most benefit.

#### Tentative explanation of the mechanisms underlying the effects of SAR218645 in models of learning and attention deficits

**Figure 12:**
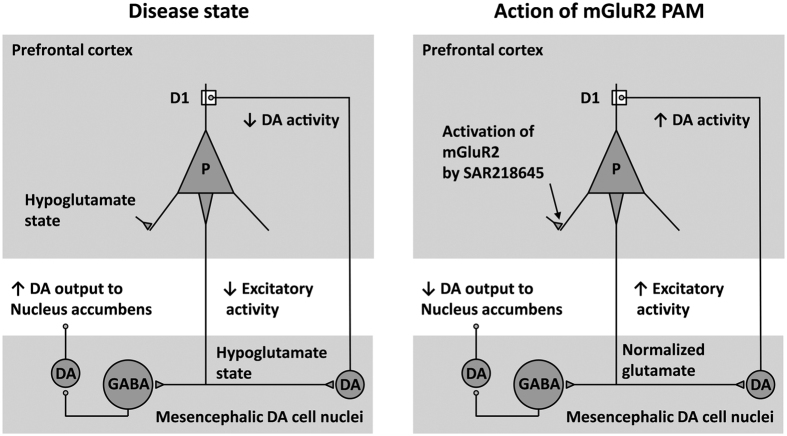
Putative mechanism underlying the effects of SAR218645 in models of learning and attention deficits in glutamatergic-dopaminergic circuits. Hypofunction of Glutamate/NMDA receptors mediating excitatory inputs to prefrontal pyramidal cells (P) in disease state leads to decreased activity in cortical excitatory projections to mesencephalic DA cell nuclei. This results in decreased activity of DA neurons projecting to the dorsolateral prefrontal cortex (DLPFC) and increased activity of DA cells projecting to the nucleus accumbens, as a consequence of decreased stimulation of GABA interneurons. SAR218645, by acting on presynaptic mGluR2 on glutamatergic nerve terminals in the prefrontal cortex, would normalize glutamatergic activity, and increase and decrease DA activity in the DLPFC and nucleus accumbens, respectively (adapted from Gruber *et al*.^90^).

The activity of SAR218645 in models predictive of activity against cognitive symptoms of schizophrenia is most likely a consequence of its capacity to affect glutamatergic tone in areas such as the prefrontal cortex, which is well known to have an essential role in executive cognitive functions, and thus directly attenuate the cognitive effects of NMDA receptor blockade or deletion as observed in NMDA Nr1^neo−/−^ mice. One could speculate that the prefrontal cortex of these latter animals was underactive, and SAR218645 thus was able to raise the developmentally induced hypoglutamatergic state. An increase of glutamate neurotransmission in the prefrontal cortex could also underlie reversal of amphetamine-induced disruption since such an increase inhibits dopamine release in the nucleus accumbens^79^. In addition, orthosteric mGluR2/3 agonists have been repeatedly found to increase dopamine levels in the prefrontal cortex^8^. Although the action of SAR218645 on the prefrontal dopaminergic function remains to be determined, it can be speculated that a functional glutamate-dopamine interaction in this area may lead to an attenuation of the amphetamine-mediated increase in subcortical dopamine tone and thus reverse the cognitive effects of amphetamine (Fig. 12). Another or additional mechanism of SAR218645’s underlying activity in cognition models may stem from findings showing that the mGluR2 plays an important role in the regulation of hippocampal cognitive functions, including induction of long term depression (LTD) of excitatory synaptic signaling (e.g. refs 80, 81, 82, 83). For example, mGluR2 knockout mice display severe deficits in LTD induced by low-frequency synaptic stimulation in hippocampal mossy fiber-CA3 synapses^83^. Moreover, studies using double mGluR2/3 knockout mice have revealed disruptions in the performance of hippocampal-dependent tasks, such as novelty preference^84^. Accordingly, it can be speculated that the effects of SAR218645 on MK-801-induced memory deficit in the object recognition test, which was demonstrated to be a nonspatial hippocampal-dependent task^85^, may involve an action at the level of the hippocampus. Equally, other potential mediators of the cognitive-improving effects of mGluR2 modulation should not be overlooked, notably norepinephrine^74^,^86^,^87^ and serotonin^74^,^88^. Finally, it is important to note that the cognitive effects in the object recognition model appeared at doses much lower than those active in the other cognitive tasks (i.e., minimal active dose: 0.01 vs. 0.1–0.3 mg/kg, po), suggesting the possibility that different mechanisms may underlie the cognitive effects of SAR218645. Taken as a whole, the findings with SAR218645 in cognitive tasks suggest that modulating allosterically the mGluR2 may offer a valuable therapeutic opportunity to alleviate certain cognitive symptoms as found in schizophrenia patients, including episodic memory impairment, attentional deficit and sensory gating disturbances.

### Investigation of potential side-effects

The collective findings from the efficacy studies in models of cognition and psychosis do not support the idea that SAR218645 may be suitable as a monotherapy for schizophrenia and instead may need to be co-administered with established antipsychotics. It is therefore important to ensure that SAR218645 does not compromise the efficacy of commonly used antipsychotics. It is also important to check that administration of SAR218645 does not induce side effects by itself, or potentiate the known side effects of established antipsychotics such as extrapyramidal symptoms. In an earlier study, SAR218645 was combined with antipsychotics in both efficacy and side effect models^89^. In the efficacy tests, SAR218645 did not alter the ability of haloperidol and risperidone to alleviate psychostimulant-induced hyperactivity in mice over a wide dose-range. Moreover, the mGluR2 PAM did not interfere with the activity of risperidone in the active avoidance test in mice. We would thus predict that combination therapy of SAR218645 with an antipsychotic would not interfere with the antipsychotic action on positive symptoms in patients with schizophrenia. In a model of extrapyramidal side-effects, SAR218645 did not induce catalepsy when given alone, and it did not modify catalepsy induced by haloperidol. Finally, as the use of orthosteric mGluR2/3 agonists has been associated with an increased risk of seizures in humans^12^, SAR218645 was tested in several mouse models of seizures. Results showed that the drug did not induce convulsant activity by itself, nor did it modify the severity of seizures following electric shocks or change the seizure threshold following PTZ administration. It is also important to note that SAR218645 did not protect from the occurrence of seizures in the maximal electroshock-induced convulsion test. Previous studies have indeed suggested that Group II mGluRs, specifically mGluR2, may be useful in the treatment of convulsive seizures^12^. However, there is no evidence from the current studies to support such an assumption.

## Conclusion

In conclusion, our studies demonstrate that the selective and orally-active mGluR2 PAM, SAR218645, shows efficacy in models relating to certain aspects of the cognitive dysfunction in schizophrenia. SAR218645 does not show potential efficacy for positive symptoms of schizophrenia alone, but does not affect the efficacy of antipsychotics when co-administered with these drugs. The drug appears safe with respect to motor side-effects and seizure activity. This preclinical profile positions SAR218645 as a promising drug candidate for the treatment of cognitive symptoms of schizophrenia patients. Moreover, in this manuscript we have tried to suggest paths forward for development of molecules such as SAR218645. Rather than a simple “one size fits all” approach to patient recruitment, the ability of SAR218645 to normalize certain endophenotypes (such as those with P50 gating abnormalities) could potentially be a way to a more targeted therapy and rational approach to initial clinical studies.

## Additional Information

**How to cite this article**: Griebel, G. *et al*. The mGluR2 positive allosteric modulator, SAR218645, improves memory and attention deficits in translational models of cognitive symptoms associated with schizophrenia. *Sci. Rep.* **6**, 35320; doi: 10.1038/srep35320 (2016).

## Supplementary Material

Supplementary Information

## Figures and Tables

**Table 1:** Pharmacokinetic parameters of SAR218645 following oral administration in mice.

Dose (mg/kg)	Tissue	Cmax (ng/mL or ng/g)	t_max_ (h)	AUC_(0-tlast)_ (hr.ng/mL or hr. hr.ng/g)	t_last_ (h)	AUC_(0-inf)_ (hr.ng/mL or hr. hr.ng/g)	T_1/2_ (h)	Brain/plasma ratio (AUC_0-inf_)
1	Plasma	131	0, 5	230	8	230	1, 5	0, 41
	Brain	32	1, 0	91	8	94	1, 5	
3	Plasma	434	0, 25	540	8	540	0, 99	0, 52
	Brain	103	1, 0	280	8	280	1, 0	
10	Plasma	1080	0, 5	2300	8	2400	1, 7	0, 42
	Brain	339	1, 0	960	8	1000	1, 6	

AUC_(0-tlast)_, area under the time-concentration curve (measured between 0 and last sampling time, t_last_); AUC_(0-inf),_ extrapolation of AUC to infinity; C_max_, maximal concentration; T_1/2_, terminal half-life; t_max_, amount of time that the drug is present at the maximum concentration in the tissue.

**Table 2:** Effects of SAR218645 on Conditioned Avoidance Response in mice.

Treatment	Dose (mg/kg, po)	Number of avoidances
Vehicle		38, 3 ± 0, 6
SAR218645	0, 01	38, 8 ± 0, 4
	0, 1	36, 6 ± 0, 8
	1	38, 4 ± 0, 3
Olanzapine	3	24, 1 ± 2, 9**

Data represent total avoidances and are expressed as mean ± S.E.M. **P < 0.01 (Dunnett’s post hoc analysis). N = 8.

**Table 3:** Effects of SAR218645, haloperidol and the combination of SAR218645 and haloperidol in the catalepsy test in rats.

Treatment	Dose (mg/kg, po)	Time (s)
Vehicle		14, 20 ± 4
Haloperidol	1	386, 2 ± 56, 08**
SAR218645	30	20 ± 5, 5
SAR218645+haloperidol	3 + 1	444, 6 ± 28, 3**
SAR218645+haloperidol	30 + 1	402, 8 ± 49, 95**

Data represent amount of time each rat spent with at least one forepaw on the bar and are expressed as mean ± S.E.M. **P < 0.01 (Dunnett’s post hoc analysis). N = 10.

**Table 4:** Effects of SAR218645 in several models of seizures in mice: comparison with the anticonvulsant levetiracetam or the benzodiazepine diazepam.

6-Hz electroshock-induced seizures	Seizure severity based on Racine’s scale
Vehicle control	3, 2 ± 0, 2
Levetiracetam 20 mg/kg ip	1, 6 ± 0, 4***
SAR218645 10 mg/kg ip	3, 5 ± 0, 2
SAR218645 30 mg/kg ip	3, 2 ± 0, 2
Pentylenetetrazol (PTZ) seizure threshold	Convulsant dose (mg/kg PTZ)
Vehicle control	41, 5 ± 1, 4
Diazepam 3 mg/kg ip	90, 7 ± 4***
SAR218645 10 mg/kg po	38 ± 0, 6
SAR218645 30 mg/kg po	38, 5 ± 0, 9
Maximal electroshock-induced convulsions	Tonic seizures (%)
Vehicle control	100
Diazepam 10 mg/kg ip	0
SAR218645 30 mg/kg ip	100

Data (6-Hz and PTZ) represent mean ± S.E.M. ***P < 0.001 versus Vehicle control (Dunnett’s test). N = 10 to 15 mice per group.

**Table 5:** Summary of the effects of SAR218645 in *in vivo* models.

Model	Species	Dose-range (mg/kg), route	Minimal active dose (mg/kg)
Functional assays			
LY404039-induced turning behavior	CD-1 mice	1–10, po	3
DOI-induced glutamate release in the medial prefrontal cortex	Sprague-Dawley rats	0.3–30, po	3
Schizophrenia			
DOI-induced head-twitch behaviors	CD-1 mice	1–10, po	3
MK-801-induced hyperactivity	Swiss mice	0.1–10, po	>10
Amphetamine-induced hyperactivity	Swiss mice	0.1–10, po	>10
Hyperactivity	NMDA Nr1^neo−/−^ mice	1–10, po	>10
Hyperactivity	DAT^−/−^ mice	3–10, po	>10
Conditioned Avoidance Response	C57BL/6J mice	0.01–1, po	>10
Novel object recognition task	Swiss mice	0.01–1, po	0.01
Y-maze test	NMDA Nr1^neo−/−^ mice	0.1, po	0.1
Amphetamine-induced disrupted latent inhibition	C57BL/6J mice	0.1–10, po	1
Amphetamine-induced disruption of auditory evoked potentials	Sprague-Dawley rats	0.3–3, po	0.3
Side-effects			
Catalepsy	Sprague-Dawley rats	30, po	>30
6-Hz-electroshock-induced seizures	OF1 mice	10–30, ip	>30
Maximal-electroshock-induced seizures	OF1 mice	30, ip	>30
Pentylenetetrazol seizure threshold	OF1 mice	10–30, po	>30
