# Supplementary material for: The mGluR2 positive allosteric modulator, SAR218645, improves memory and attention deficits in translational models of cognitive symptoms associated with schizophrenia
Source: Sci Rep. 2016 Oct 13;6:35320. doi: 10.1038/srep35320 (PMC5062470; doi:10.1038/srep35320)
Supplement: Supplementary Information [file srep35320-s1.docx]

**The mGluR2 positive allosteric modulator, SAR218645, improves memory and attention deficits in translational models of cognitive symptoms associated with schizophrenia**

Guy Griebel, Philippe Pichat, Denis Boulay, Vanessa Naimoli, Lisa Potestio, Robert Featherstone_,_ Sukhveen Sahni, Henry Defex, Christophe Desvignes, Franck Slowinski, Xavier Vigé, Olivier E. Bergis, Rosy Sher, Raymond Kosley, Sathapana Kongsamut, Mark D. Black, Geoffrey B. Varty

**Table S1**: The GPCR, ion channel, enzyme and kinase panels

| GPCRs and channels | 5-HT transporter, 5-HT_1D_, 5-HT_2A_, 5-HT_2B_, 5-HT_2C_, 5-HT_3_, 5-HT_4e_, 5-HT_6_, 5-HT_7_, A1, A2A, A3, α1A, α2A, AR, AT1, AT2, B1, B2, BB (non-selective), β1, β2, BZD (central), BZD (peripheral), Ca^2+^ channel (L, dihydropyridine site), Ca^2+^ channel (L, verapamil site) (phenylalkylamine), Ca^2+^ channel (N), Cav 3.1, CB1, CB2, CCK1 (CCKA), CCK2 (CCKB), CCR1, CCR2, Cl^-^ channel (GABA-gated), CXCR2 (IL-8B), D1, D2S, Δ2 (DOP), dopamine transporter, ERα, ETA, GABA transporter, GABAA, GABAB(1b), GAL1, GAL2, GIRK ¼, glycine (strychnine-sensitive), GR, H1, H2, H3, H4, kainate, κ (KOP), KATP channel, KV1.3, M1, M2, M3, MaxiK, MC3, MC4, MCH1, μ (MOP), N muscle-type, N neuronal α-BGTX-insensitive (α4β2), N neuronal α-BGTX-sensitive (α7), Nav 1.7 (tonic block), Nav 1.7 (frequency block), NK1, NK2, NK3, NMU2, NOP (ORL1), norepinephrine transporter, NTS1 (NT1), P2X3, P2X4, P2X7, P2Y, PCP, PR, σ (non-selective), σ1, σ2, SK4, sst (non-selective), Task1, Task3, TNF-α, TR (TH), Trek-1, TRPA1, TRPC4, TRPC5, TRPC6, TRPV1, TRPV4, TRPV6, TRPM8, V1a, V1b, V2, VPAC1 (VIP1), Y1, Y2. |
| --- | --- |
| Enzymes | 12-lipoxygenase, ACE, acetylcholinesterase, ATPase (Na^+^/K^+^), CaMK2α, cathepsin D, cathepsin L, constitutive NOS (endothelial), COX1, COX2, IRK (InsR), MAO-A, MAO-B, MMP-1, PDE3A, PDE4D, phosphatase 1B (PTP1B), PLC, tryptase. |
| Kinases | ABL1, AKT1, AKT2, AMPK, Aurora2, BTK, CAMK2D, CaMK4, CDK2-cyclinA, CHK1, CHK2, CK1d, cRAF, CTAK1, DYRK1A, ERK1, FLT2, FLT3, GSK3β, HGK, IGF1RK, INSRK, IRAK4, KDR, LCK, LynA, MAPKAPK2, MARK1, METK, MSK1, MST2, p38a, p42MAPK, p59FYN, p70S6K, PAK2, PIM2, PKA, PKCb2, PKCz, PKG1a, PRAK, PRKD2, ROCK2, RSK1, SGK, SRC, SYK. |
